# Skeleton binding protein-1-mediated parasite sequestration inhibits spontaneous resolution of malaria-associated acute respiratory distress syndrome

**DOI:** 10.1371/journal.ppat.1010114

**Published:** 2021-11-29

**Authors:** Hendrik Possemiers, Thao-Thy Pham, Marion Coens, Emilie Pollenus, Sofie Knoops, Sam Noppen, Leen Vandermosten, Sigrid D’haese, Luna Dillemans, Fran Prenen, Dominique Schols, Blandine Franke-Fayard, Philippe E. Van den Steen

**Affiliations:** 1 Laboratory of Immunoparasitology, Department of Microbiology, Immunology and Transplantation, Rega Institute for Medical research, KU Leuven, Belgium; 2 Currently at Clinical Immunology Unit, Department of Clinical Sciences, Institute of Tropical Medicine Antwerp, Belgium; 3 Laboratory of Virology and Chemotherapy, Department of Microbiology, Immunology and Transplantation, Rega Institute for Medical research, KU Leuven, Belgium; 4 Currently at Neuro-Aging & Viro-Immunotherapy (NAVI), Vrije Universiteit Brussel (VUB), Brussels, Belgium; 5 Department of Parasitology, Leiden University Medical Center, The Netherlands; McGill University, CANADA

## Abstract

Malaria is a hazardous disease caused by *Plasmodium* parasites and often results in lethal complications, including malaria-associated acute respiratory distress syndrome (MA-ARDS). Parasite sequestration in the microvasculature is often observed, but its role in malaria pathogenesis and complications is still incompletely understood. We used skeleton binding protein-1 (SBP-1) KO parasites to study the role of sequestration in experimental MA-ARDS. The sequestration-deficiency of these SBP-1 KO parasites was confirmed with bioluminescence imaging and by measuring parasite accumulation in the lungs with RT-qPCR. The SBP-1 KO parasites induced similar lung pathology in the early stage of experimental MA-ARDS compared to wildtype (WT) parasites. Strikingly, the lung pathology resolved subsequently in more than 60% of the SBP-1 KO infected mice, resulting in prolonged survival despite the continuous presence of the parasite. This spontaneous disease resolution was associated with decreased inflammatory cytokine expression measured by RT-qPCR and lower expression of cytotoxic markers in pathogenic CD8^+^ T cells in the lungs of SBP-1 KO infected mice. These data suggest that SBP-1-mediated parasite sequestration and subsequent high parasite load are not essential for the development of experimental MA-ARDS but inhibit the resolution of the disease.

## Author summary

Malaria is still a severe global disease with more than 200 million cases and 400 000 deaths each year. *Plasmodium falciparum* is the species responsible for most malaria deaths globally. The propensity of these parasites to sequester in peripheral vascular beds is assumed to play an important role in disease severity and mortality. Although sequestration has been observed in lungs of malaria patients, its role in the pathogenesis of MA-ARDS, a severe lung complication in malaria, was previously unknown. Therefore, we used sequestration-deficient SBP-1 KO *Plasmodium berghei* NK65 parasites to study the role of sequestration in experimental MA-ARDS. We observed that MA-ARDS developed similarly in WT and SBP-1 KO infected mice, but the majority of SBP-1 KO-infected mice were able to resolve the lung pathology despite the continuous presence of the parasite. This coincided with a prolonged survival, a decrease in inflammatory cytokine expression and lower expression of cytotoxicity markers in pathogenic CD8^+^ T cells. These results give important new insights in the role of parasite sequestration in malaria pathology.

## Introduction

Malaria is a severe disease caused by *Plasmodium* parasites and affects 200 million people each year, with more than 400 000 deaths [1]. Despite the availability of good antimalarial treatments and prevention measures, these numbers are stalling and both transmission and severe disease still remain in 91 countries. The symptoms of malaria range from non-lethal, like fever, headache and vomiting, to life-threatening complications, such as severe malarial anemia, cerebral malaria (CM), placental malaria and malaria-associated acute respiratory distress syndrome (MA-ARDS).

MA-ARDS is a severe lung complication with a high lethality rate, characterized by diffuse alveolar inflammation, damage to the alveolar-capillary membrane, microhemorrhages and vasogenic edema in the lungs [2]. Until now, no effective treatment is available for MA-ARDS and the precise mechanisms which lead to MA-ARDS are not completely understood. In our group, a mouse model for experimental MA-ARDS was developed. In this model, C57BL/6 mice are infected with *Plasmodium berghei* NK65 (*Pb*NK65) parasites, which cause lethal pulmonary inflammation with protein-rich interstitial and alveolar edema [3].

Parasite sequestration is thought to play an important role in malaria pathology. In patients with CM, massive parasite sequestration in the brain is associated with more severe disease and poor outcome [4,5]. Although sequestration of *Plasmodium falciparum* has also been observed in lungs of malaria patients, its role in the pathogenesis of MA-ARDS is currently unknown [6,7].

*Plasmodium falciparum* is the species responsible for most malaria deaths globally. The propensity of these parasites to sequester is assumed to play an important role in disease severity and mortality. Parasite sequestration in the vasculature is mediated by cytoadhesion of parasite adhesins expressed on the surface of infected red blood cells (iRBC) to host receptors on endothelial cells (ECs). The export of the parasite adhesins to the surface of the iRBCs is mediated by several parasite proteins, including the Maurer’s cleft skeleton binding protein-1 (SBP-1), which is both expressed in human and rodent malaria parasites [8]. Although the adhesin export system is highly conserved, the adhesins mediating sequestration of *P*. *falciparum* and *P*. *berghei* parasites are different. Sequestration of *P*. *falciparum* is mediated by *Plasmodium falciparum* erythrocyte membrane protein-1 (*Pf*EMP1) adhesins encoded by the *var* genes [9]. These *var* genes are absent in the genome of *P*. *berghei* and the adhesins mediating sequestration of *P*. *berghei* are currently unknown. SBP-1 is essential for the transport of *Pf*EMP1 adhesins to the surface of the iRBC [10,11]. *Pf*EMP1 adhesins bind to several endothelial receptors such as CD36, intercellular adhesion molecule-1 (ICAM-1), vascular cell adhesion molecule-1 (VCAM-1), platelet endothelial cell adhesion molecule-1 (PECAM-1) and chondroitin sulfate A (CSA) [12–15]. Genetic deletion of SBP-1 in *P*. *falciparum* led to the absence of *Pf*EMP1 on the surface of the iRBCs, which abolished binding to endothelial receptors [10]. CD36, ICAM-1 and VCAM-1 are also identified as important endothelial receptors for *P*. *berghei* sequestration [16–18]. Importantly, *P*. *falciparum* SBP-1 has been shown to complement the function of *P*. *berghei* SBP-1 upon SBP-1 knock-out, indicating that SBP-1 has highly similar and essential roles in the sequestration of *P*. *falciparum* and *P*. *berghei* [8].

Endothelial activation is a major hallmark of severe malaria. Biomarkers of endothelial activation can reliably predict malarial disease severity and mortality [19]. Endothelial activation leads to the increased expression of endothelial adhesion molecules and disrupts the endothelial cell–cell junctions, thereby promoting vascular leakage. The cause of endothelial activation in malaria is not fully elucidated, both sequestration and inflammation may play a role. Sequestration can cause endothelial activation by the adhesion of iRBCs itself and by the local release of components such as hemozoin, glycosylphosphatidylinositol and histones, upon schizont burst [20–24]. Furthermore, inflammatory cytokines such as IFN-γ may also play a crucial role in endothelial activation [25,26].

In this study, we investigated the role of sequestration in experimental MA-ARDS by using SBP-1 KO *Pb*NK65 parasites, which are unable to sequester, in contrast to the WT parasites. Strikingly, although WT and sequestration-deficient SBP-1 KO parasites induced similar lung pathology in the early stage of experimental MA-ARDS, the lung pathology resolved in the majority of the SBP-1 KO infected mice, resulting in prolonged survival despite the continuous presence of the parasite. This spontaneous disease resolution was never observed with WT parasites and was associated with decreased inflammatory cytokine expression and a lower expression of cytotoxic markers in pathogenic CD8^+^ T cells in lungs of SBP-1 KO infected mice.

## Results

### SBP-1 KO *Pb*NK65 parasites showed reduced sequestration compared to WT *Pb*NK65 parasites

**Fig 1:**
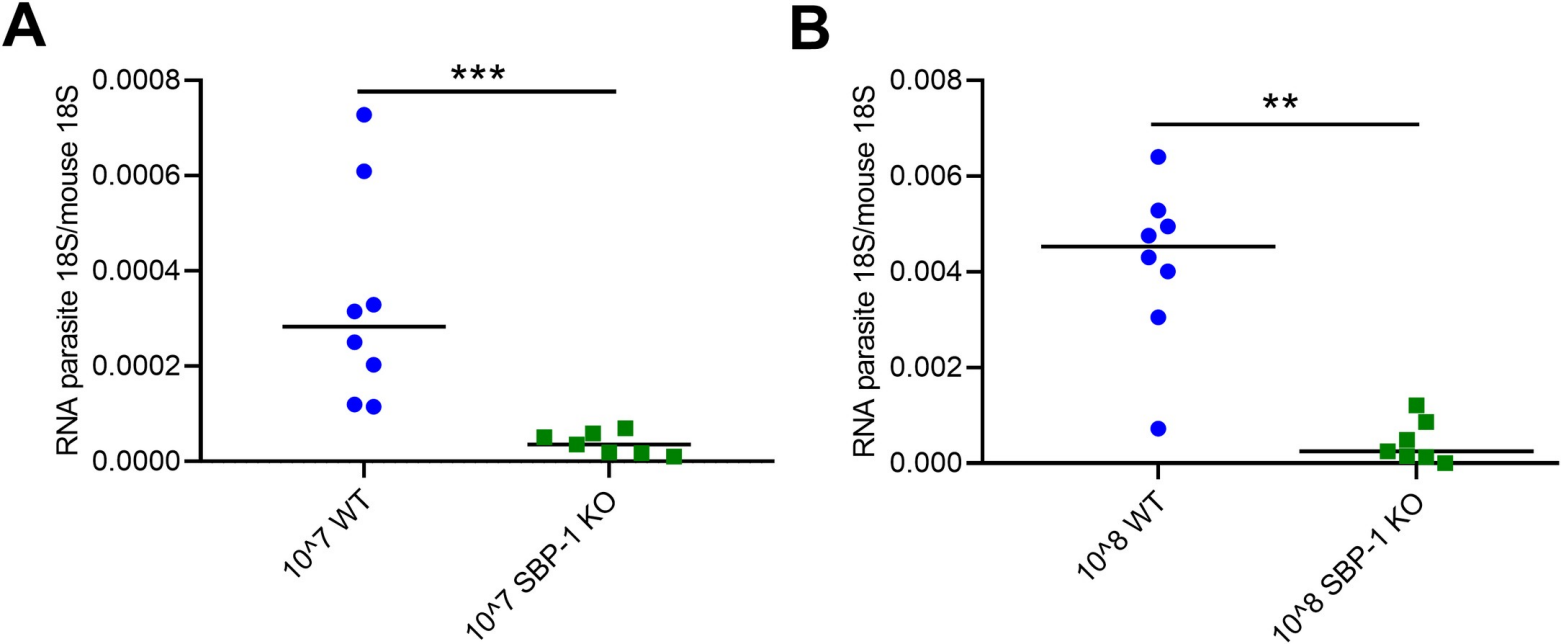
SBP-1 KO parasites sequester less than WT *Pb*NK65. Mice were i.v. injected with 10^7^ or 10^8^ SBP-1 KO or WT schizonts. After 22 h, mice were euthanized, perfused and dissected. (A-B) Lungs of the mice were homogenized and mRNA levels of parasite 18S and mouse 18S were measured with qRT-PCR. Horizontal lines with asterisks on top indicate significant differences between groups. Data of two experiments, n = 7–8 per group.

SBP-1 is essential for the transport of adhesins to the surface of the iRBC and genetic deletion of SBP-1 leads to a decreased sequestration ability [8]. To study the effect of sequestration on MA-ARDS, we generated *Pb*NK65 parasites lacking expression of SBP-1 by deleting the *sbp1* gene (PBANKA_1101300) using the same DNA construct and transfection methods as described by De Niz *et al*. for *Pb*ANKA [8], resulting in SBP-1 KO *Pb*NK65 parasites (see Materials and Methods section and S1 Fig). We first determined the differences in sequestration between WT and SBP-1 KO *Pb*NK65 parasites. Parasites were quantified in the lungs of mice by qRT-PCR 22 hours after intravenous (i.v.) injection with 10^7^ or 10^8^ WT or SBP-1 KO schizonts. Lungs were perfused to remove most of the freely circulating parasites. Importantly, this specific experimental setup avoids the influence of confounding factors, such as lung pathology, inflammation or differential host responses. A significantly higher accumulation of parasites was detected in the lungs of WT infected mice compared to the SBP-1 KO group after i.v. injection of 10^7^ and 10^8^ parasites (Fig 1A and 1B). These results clearly show that SBP-1 KO parasites sequester significantly less than WT parasites.

**Fig 2:**
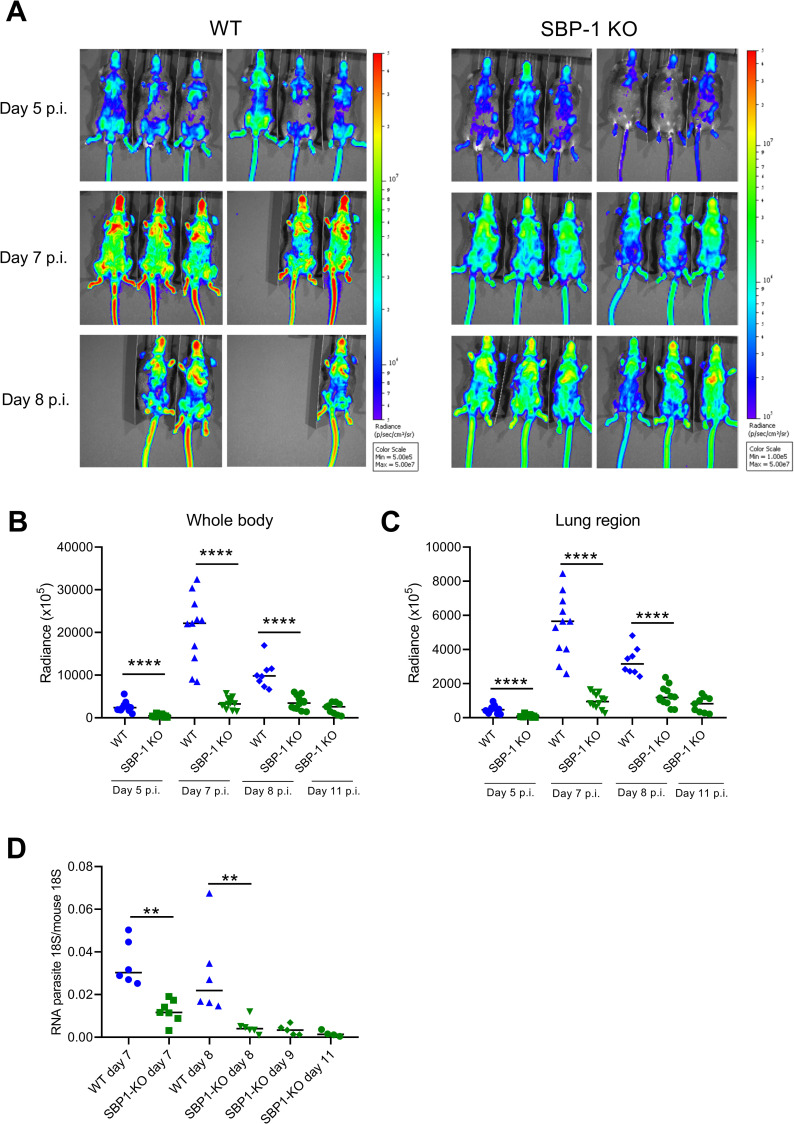
Reduced parasite accumulation in SBP-1 KO infected mice compared to WT infected mice. C57BL/6 mice were infected with WT and SBP-1 KO *Pb*NK65 parasites. (A) Whole body bioluminescent images at the indicated days p.i. of representative infected mice. Quantification of bioluminescent signals in (B) whole body and (C) lung region in WT and SBP-1 KO infected mice. Infected mice were euthanized and dissected at the indicated days p.i. and (D) lungs of the mice were homogenized and mRNA levels of parasite 18S and mouse 18S were measured with qRT-PCR. Horizontal lines with asterisks on top indicate significant differences between groups. Data of two experiments; panel A-C: n = 8–12 per group; panel D: n = 4–8 per group.

In a more conventional setup with infection of C57BL/6 mice with intraperitoneal (i.p.) injection of 10^4^ parasites, accumulation of WT and SBP-1 KO parasites were visualized in mice at different days post infection (p.i.) by *in vivo* bioluminescence IVIS imaging. The *in vivo* imaging showed a significantly higher radiance intensity in WT infected mice compared to SBP-1 KO infected mice at day 5, 7 and 8 p.i. (Fig 2A). In accordance with the known sequestration preference of *P*. *berghei* [16], we observed a significantly higher bioluminescence in whole body and lung region of WT infected mice compared to SBP-1 KO infected mice at day 5, 7 and 8 p.i. (Fig 2B and 2C). In addition, bioluminescence signals corrected for parasitemia were significantly higher in whole body and lung region at day 5 and 7 p.i. in the WT group compared to the SBP-1 KO group (S2 Fig). Furthermore, significantly higher accumulation of WT parasites compared to SBP-1 KO parasites was also observed by qRT-PCR of the lungs after perfusion at day 7 and 8 p.i. (Fig 2D). Overall, these data show that SBP-1 KO substantially reduces sequestration and parasite load of *P*. *berghei* NK65, similarly as was described for *P*. *berghei* ANKA [8].

### Survival of SBP-1 KO infected mice is significantly better than WT infected mice

**Fig 3:**
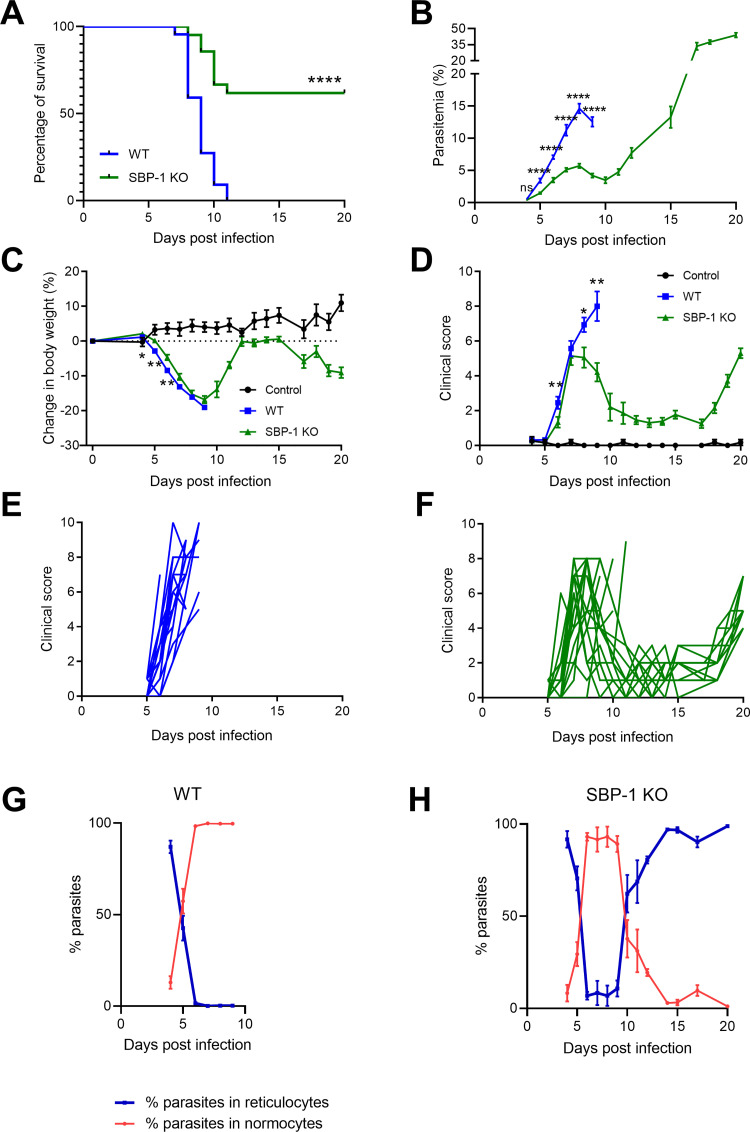
Improved survival of SBP-1 KO infected C57BL/6 mice compared with WT infected C57BL/6 mice. The disease course of WT and SBP-1 KO-infected mice was compared and (A) survival, (B) parasitemia, (C) body weight and (D-F) clinical score were monitored. Clinical score curves of (E) individual WT infected mice and (F) SBP-1 KO infected mice. (G,H) Percentages of parasites in reticulocytes and normocytes were determined at indicated time points by differential counting of blood smears. (B, C, D) Each datapoint represents mean ± SEM. Log-rank (Mantel-Cox) test was used to calculate significant differences between the survival curves. Black data lines denote uninfected control mice. Panel A-F: data of three experiments, uninfected control group: n = 6 per group, infected group: n = 21–22 per group, panel G-H: data of two experiments, n = 16 per group.

Disease course and survival of C57BL/6 mice infected with WT and SBP-1 KO *Pb*NK65 parasites were investigated. Mice infected with SBP-1 KO parasites survived significantly longer than mice infected with WT parasites. While all WT infected mice died prior to day 12 p.i., more than 60% of SBP-1 KO infected mice survived until day 20 p.i. (Fig 3A). The experiment was terminated at day 20 p.i. to avoid lethal hyperparasitemia in the surviving SBP-1 KO infected mice. Parasitemia in WT infected mice increased rapidly from day 5 to 8 p.i., while parasitemia in the SBP-1 KO infected mice was significantly lower and remained around 5% until day 12 p.i., when it started to increase rapidly with development of hyperparasitemia at day 20 p.i. (Fig 3B). The lower parasitemia with the SBP-1 KO parasite is consistent with the enhanced splenic clearance of the non-sequestering parasites, as also shown by De Niz *et al*. [8]. Despite a minor difference between WT and SBP-1 KO infected mice from day 4 to 6 p.i., body weight decrease was largely similar in both groups (Fig 3C). From day 10 to 14 p.i., body weight increased in the surviving SBP-1 KO infected mice, and decreased again after day 15 p.i. in parallel with the development of hyperparasitemia. The initial increase in clinical score at day 6 and 7 p.i. was similar with WT and SBP-1 KO parasites, but further increased at day 8 and 9 p.i. in the WT infected mice, while disease resolution was observed in the SBP-1 KO group until day 17 p.i., resulting in significant differences in the clinical score (Fig 3D–3F). From day 18 p.i. onwards, the clinical score of SBP-1 KO infected mice increased again due to hyperparasitemia. As described previously for the WT parasites, both WT and SBP-1 KO parasites show preference for reticulocytes and invade predominantly reticulocytes early during the infection (Fig 3G and 3H) [27]. Due to infection and subsequent bursting, most of the available reticulocytes were consumed and the infected reticulocytes disappeared. Hence, the parasites switched to invade normocytes from day 6 p.i. onwards (Fig 3G and 3H). Consistent with the development of anemia, new reticulocytes were released from the bone marrow in the SBP-1 KO infected mice starting from 10 days p.i., and the SBP-1 KO parasites switched back to invade reticulocytes (Fig 3H). This switch from normocytes to reticulocytes appeared two days after the significant decrease in clinical score in the SBP-1 KO infected mice, which shows that the resolution of pathology is not dependent on this switch to reticulocytes (Fig 3D and 3H). These data show that SBP-1-mediated parasite sequestration and subsequent parasite load significantly decreases survival of *Pb*NK65 infected mice, and that in the absence of parasite sequestration, the mice are able to resolve a first pathological phase of experimental MA-ARDS despite the continuous presence of the parasite.

### SBP-1-mediated sequestration is not essential for the development of pulmonary pathology but affects its resolution

**Fig 4:**
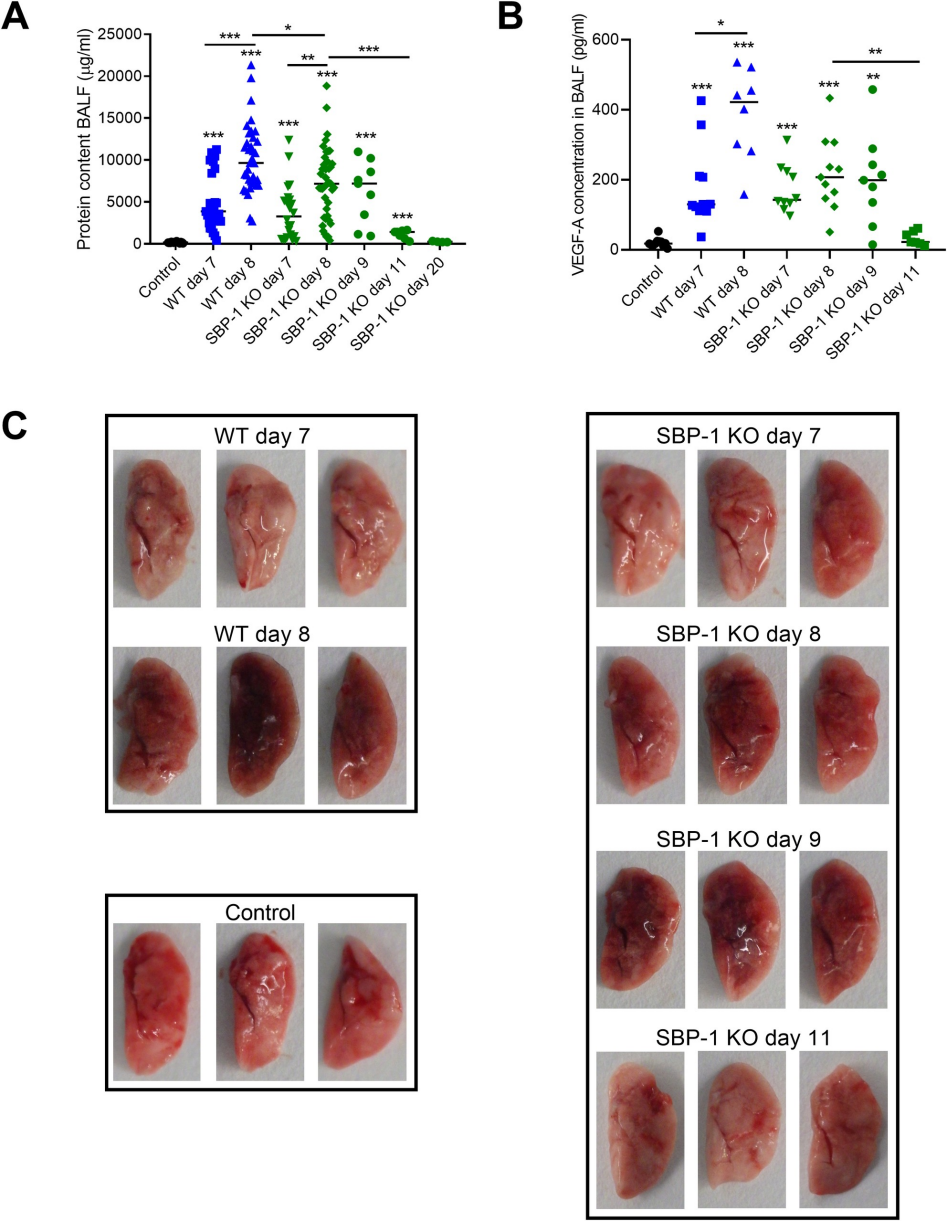
SBP-1-mediated sequestration does not affect the development of the pulmonary pathology but inhibits the resolution. C57BL/6 mice were infected with WT and SBP-1 KO *Pb*NK65 parasites. Infected mice were euthanized and dissected at day 7 and 8 p.i. (WT and SBP-1 KO), day 9, 11 and 20 p.i. (SBP-1 KO) and (A) protein content and (B) VEGF-A concentration in BALF was measured. (C) Representative pictures from non-perfused left lungs of WT and SBP-1 KO infected mice at indicated timepoints and control mice. Asterisks above data points indicate significant differences compared to control mice, asterisks above a horizontal line show significant differences between infected groups. Data of three experiments, n = 4–14 per group. To obtain a higher statistical power for small differences, the protein content in the BALF at day 7 and day 8 p.i. was analysed in 13 experiments and combined in panel A, n = 24–36. Mann-Whitney U test with Holm-Bonferroni correction for multiple testing (number of tests = 11) was performed in panel A and B.

In further experiments, we determined whether SBP-1-mediated parasite sequestration had an influence on experimental MA-ARDS by comparing the lung pathology of WT and SBP-1 KO infected mice. Alveolar edema, measured as protein content in bronchoalveolar lavage fluid (BALF), was similar at day 7 p.i. in WT and SBP-1 KO infected mice, indicating that SBP-1-mediated sequestration does not affect the early development of MA-ARDS, despite the difference in parasite load (Fig 4A). However, at day 8 p.i., a small but significant difference was observed, indicating that resolution of lung pathology already starts at day 8 p.i. in the SBP-1 KO infected mice, while further amplification of the lung pathology occurred in the WT group. Alveolar edema further improved at day 11 and day 20 p.i. in the surviving SBP-1 KO infected mice. A similar pattern was noted for lung weight, alveolar RBC numbers and the macroscopic appearance of the lungs, with a darker appearance at day 8 and 9 p.i., which corresponds to lung pathology and microhemorrhages and a less dark appearance at day 11 p.i. corresponding to resolution of lung pathology (Figs 4C and S3). Vascular endothelial growth factor-A (VEGF-A) is a multipotent molecule that induces vascular permeability and survival, migration and proliferation of ECs during angiogenesis [28]. Recently, we showed that an increase of alveolar VEGF-A is a consequence and not a cause of the pulmonary pathology in our mouse model for experimental MA-ARDS [28]. In accordance with the lung pathology, the VEGF-A concentration was significantly increased in both WT and SBP-1 KO infected mice at day 7 and day 8 p.i (Fig 4B). The significant difference in alveolar edema between WT and SBP-1 KO group at day 8 p.i. was accompanied with a trend (p = 0.0628) toward a lower VEGF-A protein concentration at day 8 p.i. in the BALF of the SBP-1 KO infected mice compared to the WT group (Fig 4A and 4B). The improved alveolar edema in the surviving SBP-1 KO infected mice at day 11 p.i. also coincided with a significant decrease in alveolar VEGF-A.

**Fig 5:**
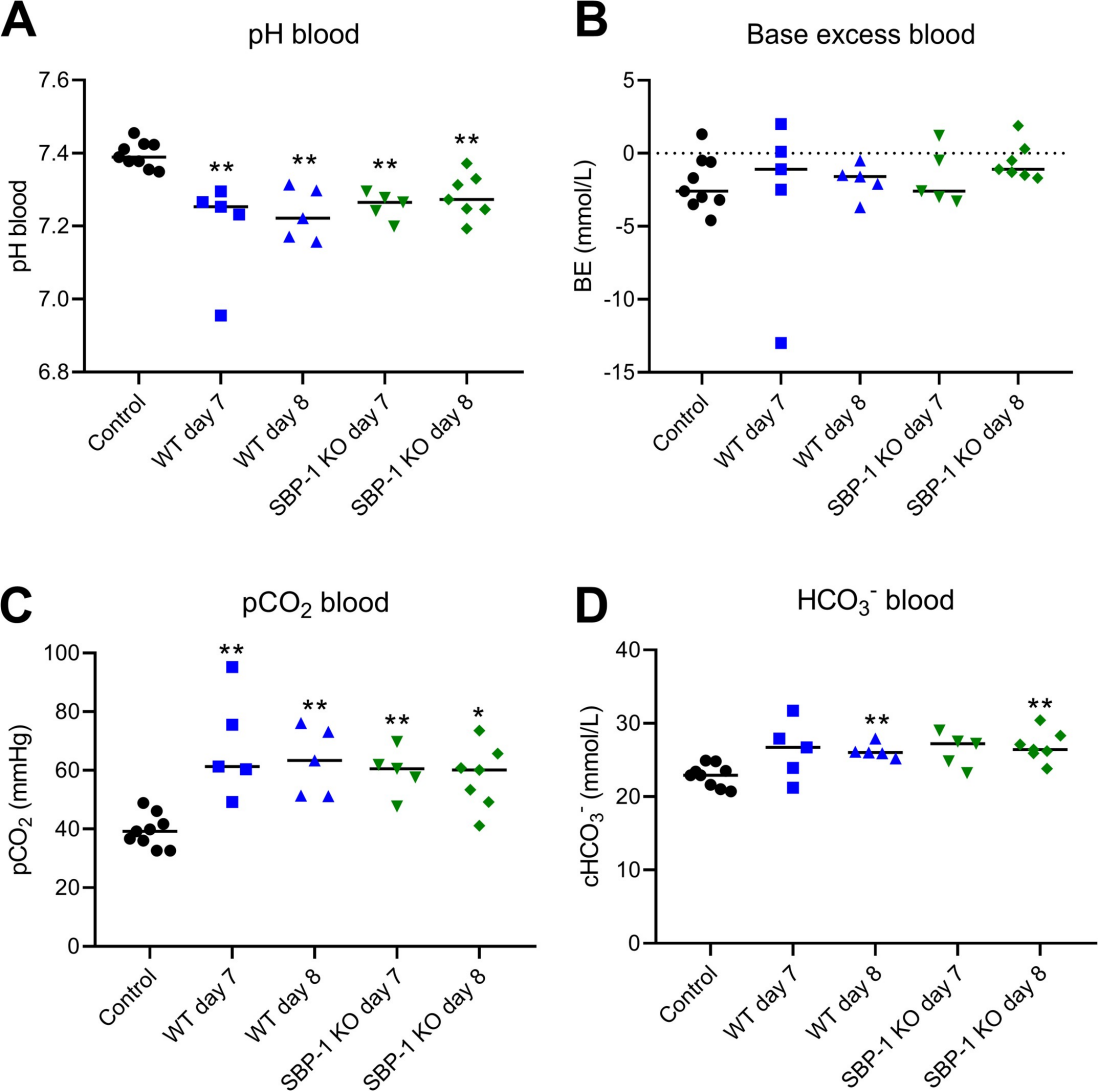
Respiratory acidosis in experimental MA-ARDS. C57BL/6 mice were infected with WT and SBP-1 KO *Pb*NK65 parasites. Retro-orbital punctures were performed and collected blood was analysed in the Epoc Blood Analysis System at indicated timepoints. (A) pH, (B) base excess, (C) partial pressure of CO_2_ and (D) HCO_3_^-^ were measured in the blood. Asterisks above data points indicate significant differences compared to control mice, asterisks above a horizontal line show significant differences between infected groups. Data of three experiments, n = 5–9 per group. Mann-Whitney U test with Holm-Bonferroni correction for multiple testing (number of tests = 8) was performed.

Furthermore, we performed blood biochemistry analyses of WT and SBP-1 KO infected mice to further characterize the consequences of the lung pathology. The pH of the blood was significantly decreased in both WT and SBP-1 KO infected mice at day 7 and 8 p.i. (Fig 5A), indicating acidosis in all infected groups. Meanwhile, the pCO_2_ and HCO_3_^-^ blood values of infected mice were increased and no decrease in base excess was observed (Fig 5B–5D). These data indicate respiratory acidosis in both WT and SBP-1 KO infected mice, which is consistent with the pulmonary pathology.

**Fig 6:**
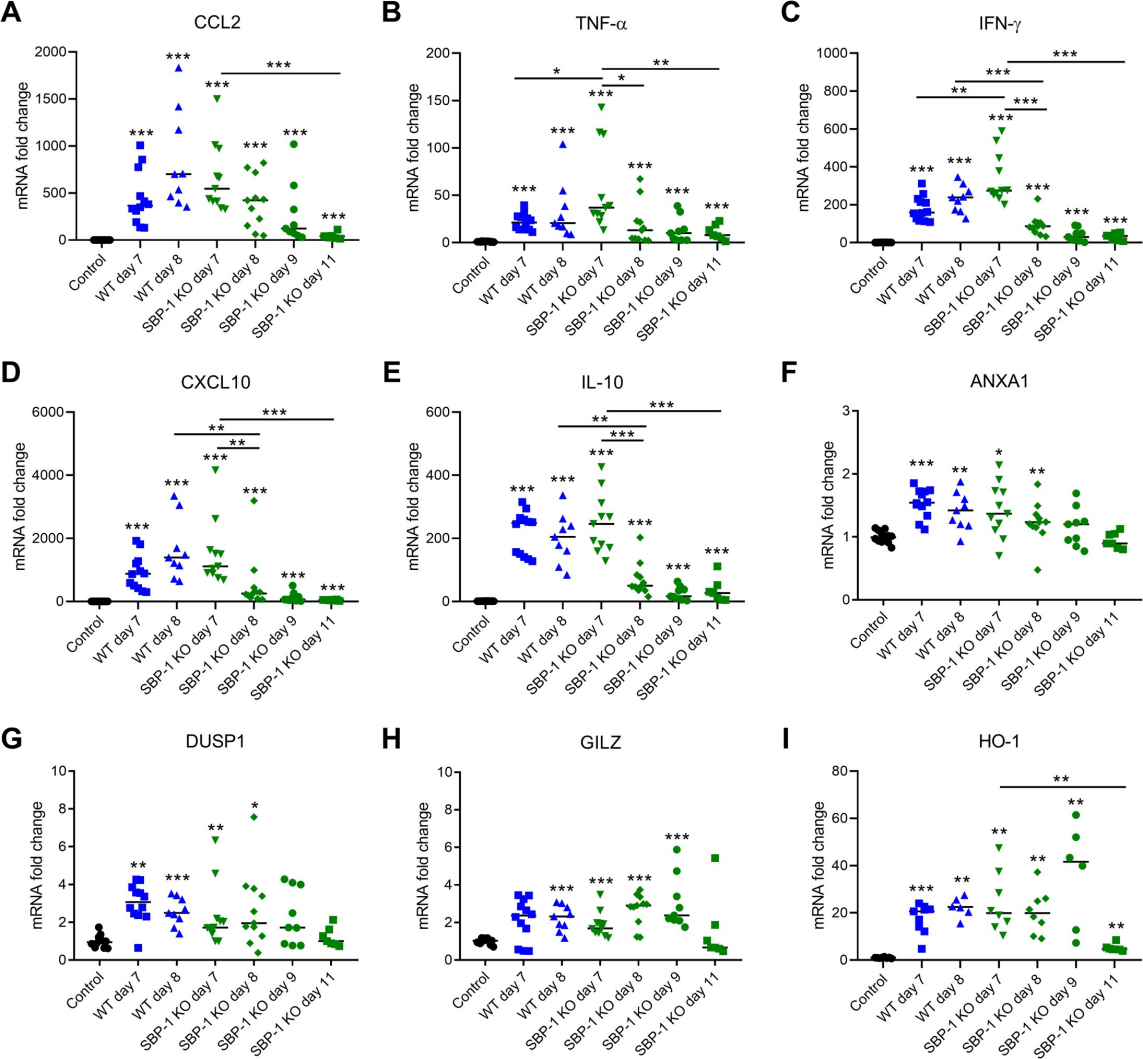
Resolution of inflammatory cytokine expression in lungs of SBP-1 KO infected mice. C57BL/6 mice were infected with WT and SBP-1 KO *Pb*NK65 parasites. Infected mice were euthanized and dissected at day 7 and 8 p.i. (WT and SBP-1 KO), day 9 and 11 p.i. (SBP-1 KO). Lungs were homogenized and mRNA levels of (A) CCL2, (B) TNF-α, (C) IFN-γ, (D) CXCL10, (E) IL-10, (F) ANXA1, (G) DUSP1, (H) GILZ, (I) HO-1 were measured by qRT-PCR. Asterisks above data points indicate significant differences compared to control mice, asterisks above a horizontal line show significant differences between infected groups. Panel A-H: data of three experiments, n = 7–12 per group, panel I: data of two experiments, n = 6–9 per group. Mann-Whitney U test with Holm-Bonferroni correction for multiple testing (number of tests = 11) was performed.

Since excessive pulmonary inflammation is also present in experimental MA-ARDS [3], we compared the lung inflammation in both groups of infected mice by measuring mRNA levels of different cytokines and chemokines. At day 7 p.i., a significantly higher expression of TNF-α and IFN-γ was observed in the SBP-1 KO infected group compared to the WT infected group, while CCL2 and CXCL10 expression remained the same (Fig 6A–6D). In the WT group, the expression levels of the different cytokines and chemokines remained at a similar level from day 7 to day 8 p.i. (Fig 6A–6D). In contrast, a significant decrease of TNF-α, IFN-γ and CXCL10 expression was observed in the SBP-1 KO group. This decreasing trend was observed until day 11 p.i. and further corroborates that resolution of the experimental MA-ARDS starts from day 8 p.i. in mice infected with the sequestration-deficient parasites. The expression of the anti-inflammatory molecules IL-10, ANXA1, DUSP1, GILZ and HO-1 was significantly increased upon infection (Fig 6E–6I). However, the expression of these molecules was not different between WT and SBP-1 KO infected mice, suggesting that these anti-inflammatory markers may be not responsible for the decrease in inflammatory cytokines at 8 days p.i. in the SBP-1 KO infected mice.

### SBP-1-mediated sequestration does not affect endothelial activation in the lung

**Fig 7:**
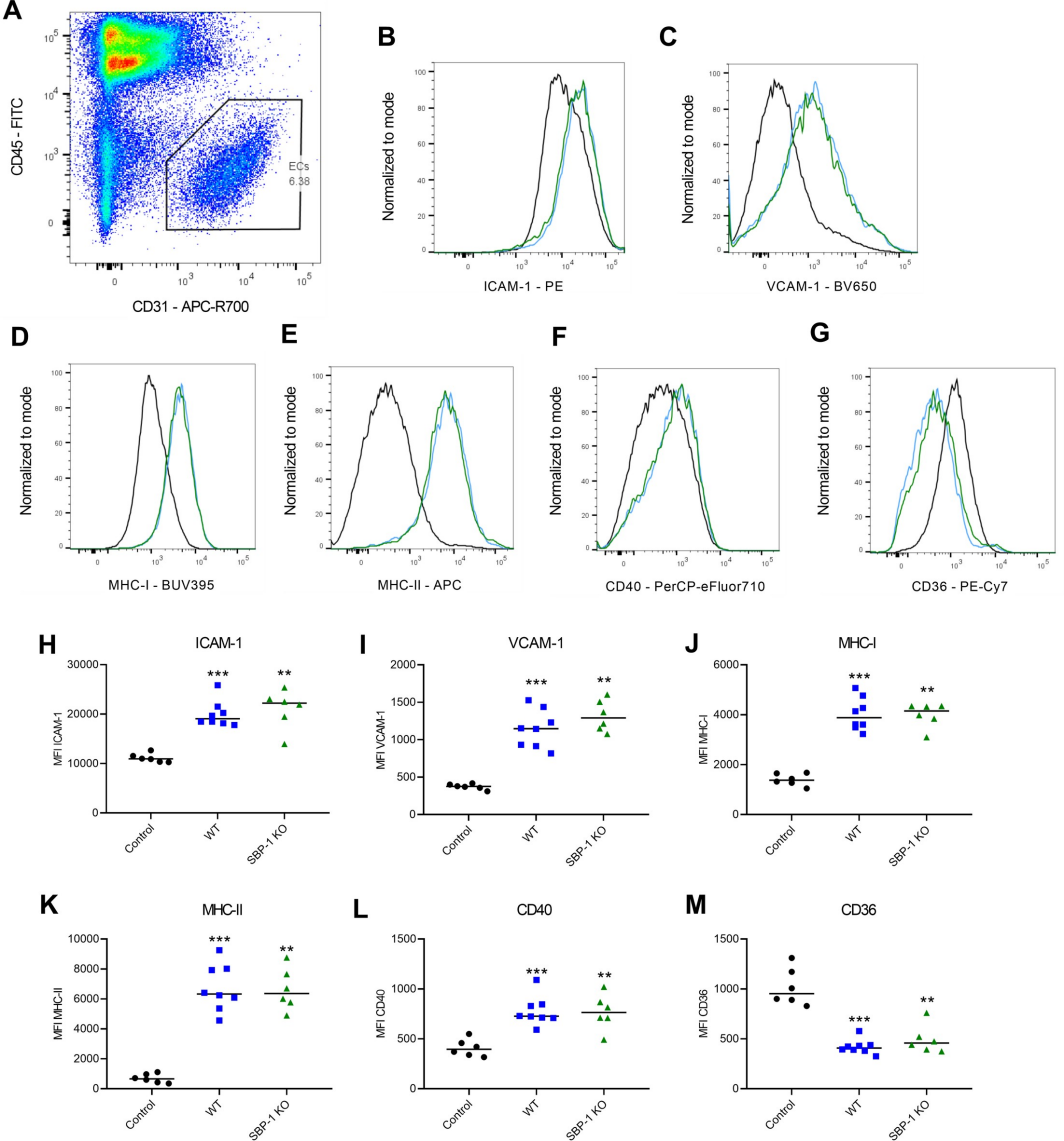
Similar expression of endothelial activation markers on pulmonary ECs in WT and SBP-1 KO infected mice. C57BL/6 mice were infected with WT and SBP-1 KO *Pb*NK65 parasites. (A) Representative flow cytometry plot of lung ECs gating. Histograms of (B) ICAM-1, (C) VCAM-1, (D) MHC-I, (E) MHC-II, (F) CD40 and (G) CD36 expression at day 8 p.i. on lung ECs in control mice (black) and WT (blue) and SBP-1 KO (green) infected mice. Mean fluorescent intensity (MFI) of (H) ICAM-1, (I) VCAM-1, (J) MHC-I, (K) MHC-II, (L) CD40 and (M) CD36 at day 8 p.i. on lung ECs. Asterisks above data points indicate significant differences compared to control mice, asterisks above a horizontal line show significant differences between infected groups. Data of two experiments, n = 6–8 per group.

Endothelial activation plays an important role in malaria pathogenesis. Biomarkers of endothelial activation reliably predict disease severity and outcome. The *in vivo* role of parasite sequestration in endothelial activation has not been fully elucidated, although *in vitro* studies have suggested that sequestering parasites may cause endothelial activation [29]. Therefore, we further characterized endothelial activation in lungs at 8 days p.i. with WT and SBP-1 KO parasites. We measured the expression levels of various markers of endothelial activation on lung ECs with flow cytometry. Significantly higher expression of ICAM-1, VCAM-1, MHC-I, MHC-II and CD40 was observed in both WT and SBP-1 KO infected mice at day 8 p.i. compared to the control mice (Fig 7B–7M). However, the expression of these endothelial activation markers was similar in both infected groups. The expression of CD36, a cell surface scavenger receptor which is also an important receptor for *P*. *berghei* parasite sequestration in the lungs, was also measured [16]. The endothelial expression of CD36 was significantly downregulated in all infected mice compared to the control mice, but no difference was observed between WT and SBP-1 KO infected mice (Fig 7G and 7M). These data suggest that sequestration deficiency, lower parasite load and early disease resolution with the SBP-1 KO parasites have no influence on the endothelial activation at day 8 p.i. in the lungs.

### Comparable pulmonary leukocyte numbers in lungs of WT and SBP-1 KO infected mice

**Fig 8:**
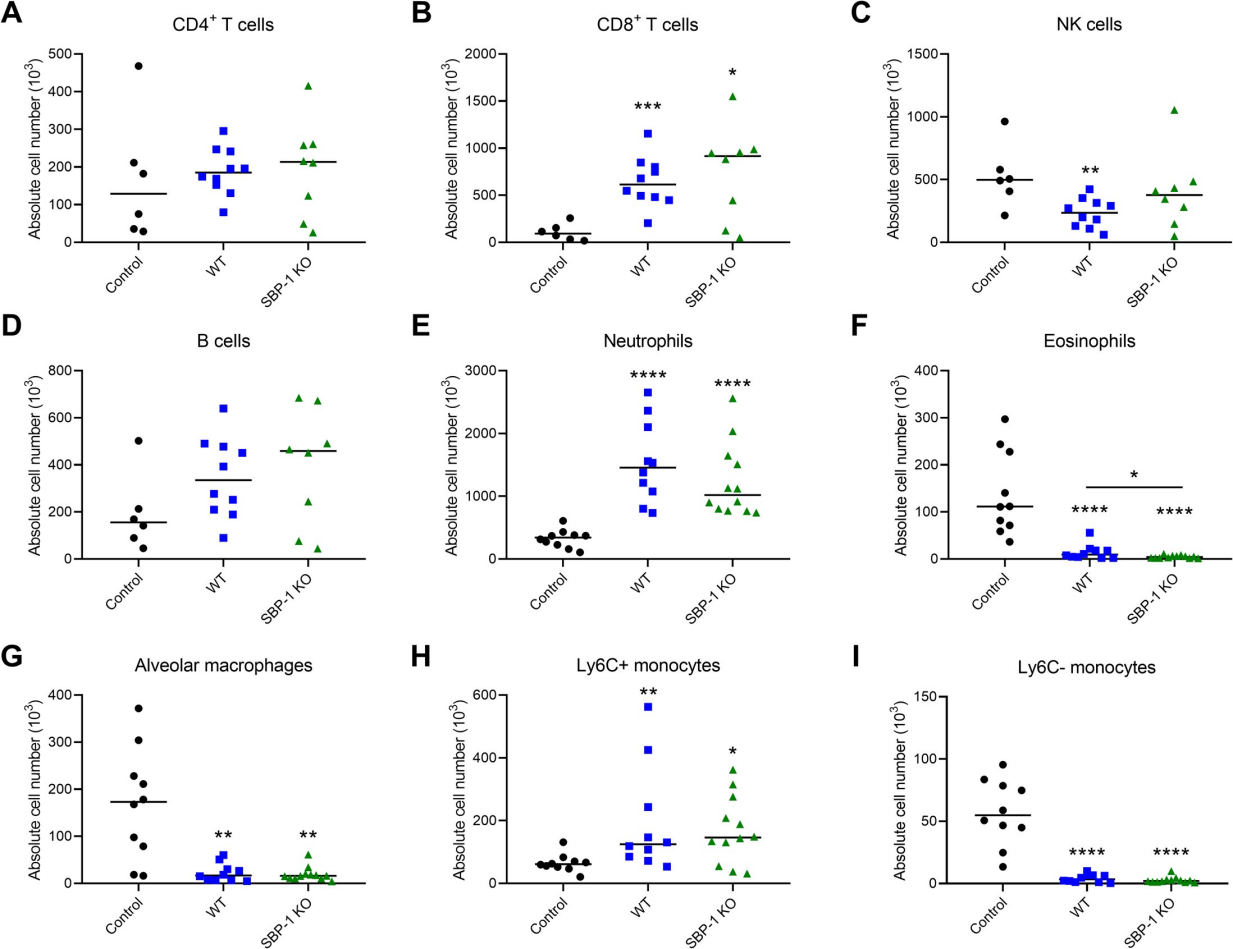
Comparable cell counts of lymphoid and myeloid subsets in lungs of WT and SBP-1 KO infected mice. Flow cytometric analysis at day 8 p.i. of pulmonary lymphoid and myeloid cells. Leukocytes (CD45^+^) were subdivided in (A) CD4^+^ T cells (CD3^+^ NK1.1^-^ CD4^+^), (B) CD8^+^ T cells (CD3^+^ NK1.1^-^ CD8^+^), (C) NK cells (CD3^-^ NK1.1^+^), (D) B cells (CD3^-^ NK1.1^-^ B220^+^), (G) alveolar macrophages (Siglec F^+^ CD11c^+^). Myeloid cells (CD3^-^ CD19^-^ NK1.1^-^) were subdivided in (E) neutrophils (Ly6G^+^ CD11b^+^), (F) eosinophils (Siglec F^+^ CD11c^-^), (H) Ly6C^+^ monocytes (CD11b^+^ MHCII^-^ Ly6C^+^) and (I) Ly6C^-^ monocytes (CD11b^+^ MHCII^-^ Ly6C^-^). Absolute cell numbers for whole lungs are shown. Asterisks above data points indicate significant differences compared to control mice, asterisks above a horizontal line show significant differences between infected groups. Data of two experiments, n = 6–12 per group.

To determine whether early disease resolution influenced the leukocyte recruitment towards the lungs of mice with experimental MA-ARDS, several immune cell subsets were analysed at day 8 p.i. with flow cytometry. CD4^+^ T cell and B cell numbers were similar in the infected and control mice (Fig 8A and 8D). NK cell numbers were only significantly decreased in the WT group compared to the control group, however no difference in the number of NK cells was observed between WT and SBP-1 KO infected mice (Fig 8C). CD8^+^ T cells, neutrophils and Ly6C^+^ monocytes were similarly increased in WT and SBP-1 KO infected mice (Fig 8B, 8E and 8H). Alveolar macrophages and Ly6C^-^ monocytes were similarly decreased in both infected groups compared to the control group (Fig 8F, 8G and 8I). The decrease in eosinophils was significantly more pronounced in the SBP-1 KO infected mice compared to the WT infected mice (Fig 8F). In summary, these data indicate that there were no major differences in pulmonary leukocyte numbers between both infected groups, apart from the eosinophils.

Since recruited leukocytes in lungs of infected mice may originate from the spleen, several immune cell subsets of the spleen were analysed at day 8 p.i. by flow cytometry. The spleen weights of the SBP-1 KO infected mice were significantly increased compared to control and WT infected mice (S4 Fig). The cell numbers of most leukocyte subsets were significantly decreased in the infected mice compared with control mice, with significantly higher numbers in SBP-1 KO infected mice compared to WT infected mice (S4 Fig).

### SBP-1 deficiency is associated with lower perforin, granzyme B and IFN-γ and higher KLRG1 expression in CD8^+^ T cells

**Fig 9:**
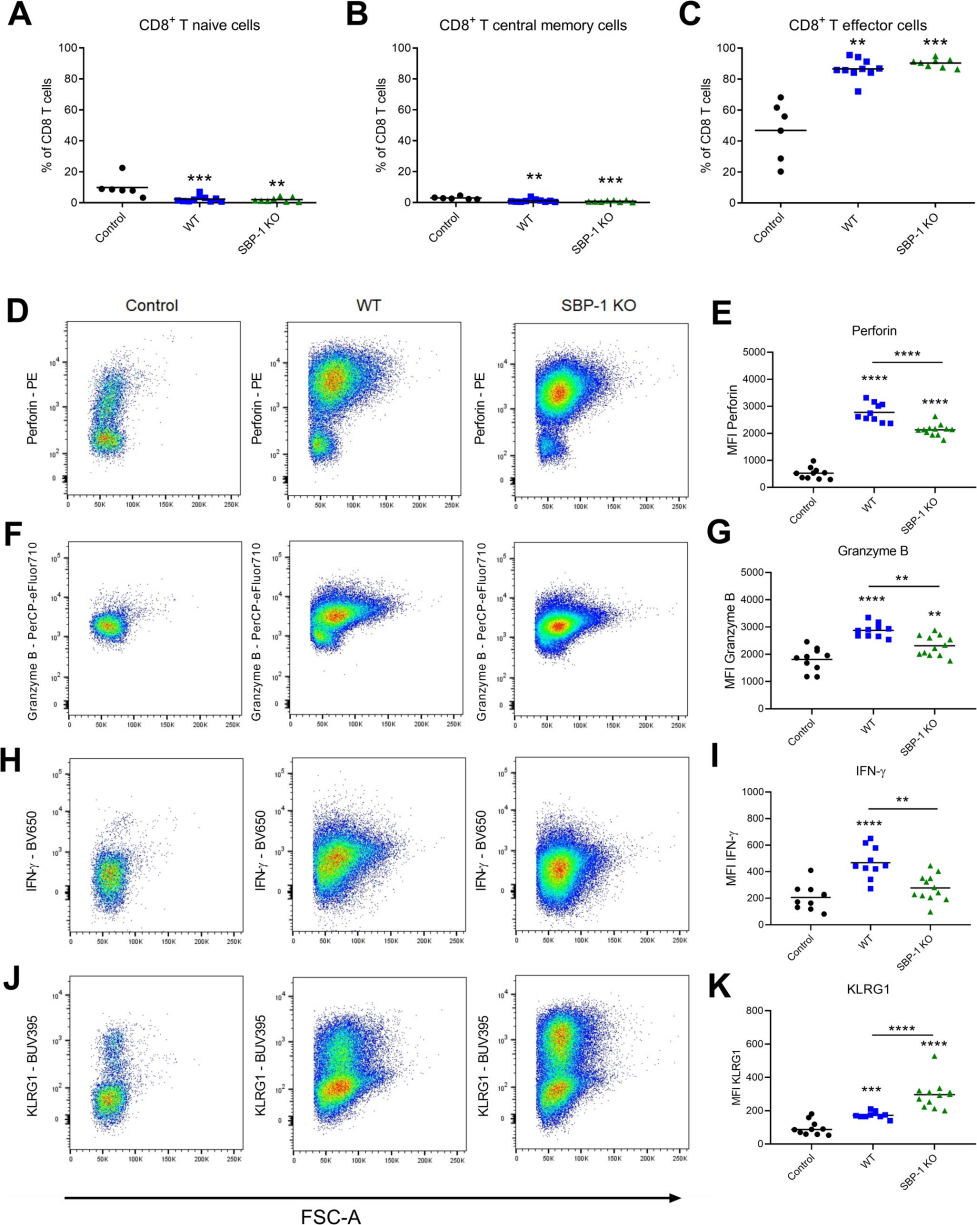
Lower expression of cytotoxic markers and higher expression of KLRG1 in pulmonary CD8^+^ T cells of SBP-1 KO infected mice. Percentages (A) CD8^+^ T naïve cells (CD62L^-^ CD44^-^) (B) CD8^+^ T central memory cells (CD62L^+^ CD44^+^) (C) CD8^+^ T effector cells (CD62L^-^ CD44^+^) of total CD8^+^ T cells. Representative flow cytometry plots show expression of (D) perforin, (F) granzyme B, (H) IFN-γ and (J) KLRG1 in CD8^+^ T cells in lungs of control mice and at 8 days p.i. with WT and SBP-1 KO parasites. Mean fluorescent intensity (MFI) of (E) perforin, (G) granzyme B, (I) IFN-γ and (K) KLRG1 of CD8^+^ T cells. Asterisks above data points indicate significant differences compared to control mice, asterisks above a horizontal line show significant differences between infected groups. Data of two experiments, n = 6–12 per group.

Although no differences in CD8^+^ T cells numbers were observed in the lungs, we further characterized CD8^+^ T cells because of their essential pathogenic role in experimental MA-ARDS [3]. The naïve, effector and central memory CD8^+^ T cells subsets and the cytotoxicity markers of the CD8^+^ T cells were characterized at day 8 p.i. in lungs of WT and SBP-1 KO infected mice. Infection resulted in higher proportions of effector CD8^+^ T cells, whereas no differences in these CD8^+^ T cell subsets were observed between WT and SBP-1 KO group (Fig 9A–9C). However, a significantly higher expression of perforin, granzyme B and IFN-γ at day 8 p.i. was observed in pulmonary CD8^+^ T cells of the WT group compared to the SBP-1 KO group, suggesting that the initiation of disease resolution in SBP-1 KO-infected mice is associated with a lower cytotoxicity of these cells (Fig 9D and 9E). Comparable findings were observed for granzyme B and IFN-γ expression in splenic CD8^+^ T cells, in particular when focusing on the effector cells (S5 Fig).

The expression of the inhibitory receptor KLRG1 was higher on the pulmonary CD8^+^ T cells in the SBP-1 KO group compared to the WT group (Fig 9J and 9K). This KLRG1 expression was negatively correlated with perforin and granzyme B, but not IFN-γ expression, in pulmonary CD8^+^ T cells of WT and SBP-1 KO infected mice (S6 Fig). KLRG1 expression was also significantly higher in the CD8^+^ T effector cells in the spleen of the SBP-1 KO infected mice compared to the WT infected mice (S5 Fig). Overall, SBP-1 deficiency was thus associated with lower expression of perforin, granzyme B and IFN-γ and higher expression of KLRG1 by CD8^+^ T cells.

## Discussion

Parasite sequestration is thought to play an important role in malaria pathology. However, the contribution of parasite sequestration and subsequent parasite load in the development of MA-ARDS pathology is not clear. In this study, we show that parasite sequestration had no influence on the development of experimental MA-ARDS. However, SBP-1-mediated sequestration did affect the resolution of MA-ARDS, as indicated by the survival of the majority of the mice infected with SBP-1-deficient parasites. Claser and colleagues also observed that vascular leakage in lungs of *Pb*ANKA infected C57BL/6 mice did not correlate with the amount of locally sequestering parasites. They demonstrated that iRBC accumulation reached a plateau at day 5 p.i. while vascular leakage was not yet observed [26]. In contrast, in the same mouse model Lovegrove *et al*. showed that CD36-dependent parasite sequestration affected the accumulation of IgM in BALF as a marker for the development of acute lung injury (ALI) [30]. However, CD36 deficiency may also affect the production of IgM [31]. Furthermore, CD36 also plays a significant role in proinflammatory cytokine responses and Th1 development, which is important for controlling parasite burden [32]. Hence, the decreased alveolar IgM accumulation observed in the CD36 KO mice could also be caused by a decreased inflammatory response in the absence of CD36 [30]. Our data also indicate a decreased expression of CD36 on pulmonary ECs in the infected mice, with a similar decrease in WT and SBP-1 KO infected mice.

The experimental setup in Fig 1, with injection of large amounts of schizonts and performing the analysis 22h later, avoids confounding factors such as differential immune responses and shows the crucial role of SBP-1 in sequestration. When infecting the mice according to our classical protocol including daily follow-up during 8 to 11 days, we observed a significantly lower parasite accumulation in the whole body with bioluminescence imaging in the SBP-1 KO infected mice compared to WT infected mice. After correction for peripheral parasitemia, the bioluminescence signals were still significantly lower in whole body and lung region at day 5 and 7 p.i., further suggesting a higher parasite accumulation/sequestration in the tissues compared to the circulation (S2 Fig). At day 8 p.i., the difference of the bioluminescence signal was less pronounced upon correction for the parasitemia. The difference between WT and SBP-1 KO group at this time may have been blurred by e.g. leukocytes, which could contribute to parasite accumulation. The parasitemia in the SBP-1 KO infected mice was also significantly lower in the early infection (until day 9 p.i.). This was also observed by De Niz *et al*., which was consistent with the increased clearance of the non-sequestering SBP-1 KO parasites [8]. These data show an overall reduced parasite load in the SBP-1 KO infected mice. In patients with malaria the estimated total parasite load was clearly associated with increased disease severity and worsened outcome [33]. Thus, the lower parasite load in the SBP-1 KO infected mice may influence survival and resolution alone or in combination with the decreased parasite sequestration in the lungs. Meanwhile, the higher parasite sequestration and parasite load with the WT parasites may have contributed to the amplification of the lung pathology. With the current approach, it was not possible to discriminate between the direct effects of parasite sequestration versus parasite load or a combination of both as cause of the observed phenotype. The lower parasite load observed with the SBP-1 KO parasite is fully consistent with a higher parasite clearance by the spleen, although we cannot fully exclude minor differences in intrinsic growth rate between WT and SBP-1 KO parasites.

Our finding that experimental MA-ARDS develops in mice infected with both WT and sequestration-deficient SBP-1 KO *Pb*NK65 is consistent with the observation that MA-ARDS in humans can be caused by both *P*. *falciparum* and *P*. *vivax*, while *P*. *vivax* sequesters to a lesser extent than *P*. *falciparum* [2,34]. Moreover, in experimental MA-ARDS parasite sequestration and its subsequent higher parasite load with WT *Pb*NK65 were associated with a higher lethality than with the SBP-1 KO *Pb*NK65. Similarly, MA-ARDS with *P*. *falciparum* has typically a worse prognosis than with *P*. *vivax* [35]. In analogy with our data, we hypothesize that spontaneous disease resolution may occur more easily with *P*. *vivax*, while the sequestration of *P*. *falciparum* may inhibit this resolution. However, we cannot exclude that the worse prognosis of MA-ARDS with *P*. *falciparum* may result from the simultaneous occurrence of other complications.

Our findings indicate the initial lung pathology at 7 days p.i. largely disappears in mice infected with SBP-1 KO parasites, as indicated e.g. by the alveolar edema measurements. Therefore, this resolution could be considered ‘spontaneous’, because the pathology heals without any treatment. MA-ARDS is a Th1 mediated pathology but the mechanisms of resolution in such pathologies are poorly understood. We recently developed a model to study resolution in experimental MA-ARDS, by treating *Pb*NK65 WT infected mice with artesunate and chloroquine, which leads to resolution of MA-ARDS in more than 80% of the mice [36]. In that model, the treated mice had a decrease in clinical score and alveolar edema and an increase in body weight, similar to the surviving SBP-1 KO infected mice in our current study. However, an important difference between the anti-malarial induced resolution and the spontaneous resolution is the concurrent presence of parasites during resolution in the SBP-1 infected mice. Other than the CCR2-independency of the antimalarial treatment-induced resolution [36], the resolution mechanisms of experimental MA-ARDS remain currently unknown. Since it is currently well-recognized that disease resolution and healing are highly active processes, it remains of paramount importance to determine the resolution mechanisms in this pathology [37].

As a further corroboration, we show for the first time respiratory acidosis in this mouse model for experimental MA-ARDS, which was also sequestration-independent. Respiratory acidosis is associated with an increased PaCO_2_ and decreased blood pH. In a study with Vietnamese adult patients with severe *falciparum* malaria, two patients had compensated respiratory acidosis with an increased PaCO_2_ and a normal arterial pH, and one patient exhibited a mixed respiratory and metabolic acidosis [38]. Patients infected with *P*. *falciparum* with MA-ARDS had increased PaCO_2_ levels and decreased blood pH, which indicates respiratory acidosis [39,40]. Respiratory acidosis was also observed in a MA-ARDS patient infected with *P*. *vivax* [41]. These observations further support the similarity of our mouse model to human MA-ARDS.

In the current study, we demonstrate that sequestration is not the main driver of the pulmonary pathology, in contrast to the inflammatory response [26,28,42]. The inflammatory response in the lungs was similar at day 7 p.i. in the WT and SBP-1 KO group and started to resolve at day 8 p.i. in the SBP-1 KO infected mice. In particular, the expression of several inflammatory cytokines decreased significantly from day 7 to day 8 p.i. in the SBP-1 KO infected mice. Claser *et al*. showed that pulmonary ECs cross-present malarial antigens on MHC-I and parasite antigen-specific CD8^+^ T cells recognize these pulmonary cross-presenting ECs and eventually cause lung pathology [26]. Assuming that this is effectively the main cause of disruption of the alveolar-capillary membrane, it is thus interesting to note that parasite sequestration seems not required for the cross-presentation of these parasite antigens, as similar pathology develops with the SBP-1 KO parasite.

The characterization of ECs in this study demonstrated that the expression of several endothelial activation markers was significantly increased after infection but no difference between WT and SBP-1 KO infected mice was observed. Dose-dependent increase in ICAM-1 expression was also observed in an *in vitro* study, in which human microvascular ECs were stimulated with *P*. *falciparum* parasites [29]. Meanwhile, we observed a similar expression of ICAM-1 on pulmonary ECs in WT and SBP-1 KO infected mice, despite the significantly lower parasite load in lungs of the SBP-1 KO infected mice. This suggests that sequestration is not the main driver of endothelial activation *in vivo*.

Interestingly, while the endothelial activation in the lung was similar at day 8 p.i. in the WT and SBP-1 KO infected mice, the pulmonary expression of various inflammatory cytokines already decreased in the SBP-1 KO infected mice. This may apparently contradict the crucial role of IFN-γ in the endothelial activation, but Valenzuela *et al*. described that ICAM-1 protein expression can persist for up to 21 hours after termination of IFN-γ stimulation [43]. Similarly, Vanhee *et al*. showed that ICAM-1 persisted at elevated levels at the cell surface for up to 5 days in human umbilical vein endothelial cells (HUVEC) after treatment with TNF-α [44]. Therefore, this apparent contradiction may result from the persisting expression of the endothelial activation markers after cytokine stimulation.

Furthermore, we observed that the expression of perforin, granzyme B and IFN-γ was higher in the pulmonary CD8^+^ T cells of the WT infected mice compared to the SBP-1 KO infected mice. Comparable findings were observed for granzyme B and IFN-γ expression in splenic CD8^+^ T cells, mainly in the effector cells, which shows that these differences are not lung specific. Claser *et al*. found a significant increase in parasite-specific CD8^+^ T cells in the lungs of *Pb*ANKA infected C57BL/6 mice, which expressed high levels of IFN-γ and granzyme B [26]. They also observed these activated CD8^+^ T cells in both lungs and spleen. In addition, the expression of KLRG1 was significantly increased on the pulmonary CD8^+^ T cells of the SBP-1 KO infected mice compared to the WT infected mice. KLRG1 is an inhibitory receptor expressed on NK cells and antigen-experienced T cells [45]. Robbins *et al*. showed that the engagement of KLRG1 inhibited IFN-γ and TNF-α production [46]. Furthermore, *in vitro* experiments demonstrated that NK cells expressing KLRG1 had a lower cytotoxicity against target cells [47]. Similarly, in our study the increased expression of KLRG1 was associated with a lower expression of the cytotoxic markers and IFN-γ in the pulmonary CD8^+^ T cells of the SBP-1 KO infected mice. This suggests a potential role of KLRG1 in decreasing the cytotoxicity of the CD8^+^ T cells in experimental MA-ARDS. Similarly as in experimental MA-ARDS, CD8^+^ T cells play an important pathogenic role in experimental cerebral malaria (ECM) [48]. ECM pathology is dependent on perforin and granzyme B, demonstrating the important contribution of these cytotoxic markers to pathology [49,50]. Recently, Riggle *et al*. observed that CM in children was associated with cerebrovascular engagement of CD8^+^ T cells expressing granzyme B [51]. Despite the fact that the role of CD8^+^ T cells in human MA-ARDS remains to be proven, these findings show the potential role of these cells in the pathogenesis of human malaria complications.

In conclusion, we describe in this study the surprising finding that although SBP-1-mediated parasite sequestration and subsequent parasite load does not contribute to the early development of MA-ARDS, it plays an important role by inhibiting disease resolution and thereby drastically increases the mortality. These data therefore contribute significantly to the understanding of MA-ARDS, and indicate that improved disease resolution may be a highly important target to decrease the unacceptably high mortality of complicated malaria.

## Materials and methods

### Ethics statement

All experiments at the KU Leuven were performed according to the regulations of the European Union (directive 2010/63/EU) and the Belgian Royal Decree of 29 May 2013, and were approved by the Animal Ethics Committee of the KU Leuven (License LA1210251, project P196/2015 and license LA1210186, project P052/2020, Belgium).

The experiments for the generation of the SBP-1 KO *Pb*NK65 parasites were performed at the Leiden University Medical Center with a licence by Competent Authority after an advice on the ethical evaluation by the Animal Experiments Committee Leiden (DEC 10099). All experiments were performed in accordance with Experiments on Animals Act (Wod, 2014), the applicable legislation in the Netherlands in accordance with the European guidelines (EU directive no. 2010/63/EU) regarding the protection of animals used for scientific purposes. All experiments were executed in a licensed establishment for the use of experimental animals.

### Parasites and mice

Equal numbers of male and female C57BL/6 mice were obtained from Janvier (7–8 weeks old, Le Genest-Saint-Isle, France). All mice were housed in individually ventilated cages in a 12 h light and 12 h dark cycle in SPF animal facility. Drinking water was supplemented with 4-amino benzoic acid (0.375 mg/ml PABA, Sigma-Aldrich, Bornem, Belgium). Mice were infected with *P*. *berghei* NK65 2168cl2 (WT) [28] or sequestration-deficient *P*. *berghei* NK65 2559cl2 (SBP-1 KO, see below) parasites by i.p. injection of 10^4^ iRBCs as described in Van den Steen *et al*. [3]. Mice were euthanized with Dolethal (Vétoquinol, Aartselaar, Belgium; 200 mg/mL, i.p. injection of 100 μL) at indicated time points.

Female OF1 mice (6–7 weeks; Charles River, NL) were used for the experiments for the generation of the SBP-1 KO *Pb*NK65 parasites. These mice were housed in individually ventilated cages furnished with autoclaved aspen woodchip, fun tunnel, wood chew block and nestlets at 21 ± 2°C under a 12 h light and 12 h dark cycle. All experiments involving generation of knock-out and cloning of parasite lines were performed using highly standardized and approved protocols that have been developed to reduce the number of animals and minimize suffering and distress. In all experiments mice were killed at a parasitemia of 2–5% before malaria-associated symptoms occur. Mice were killed either by cardiac puncture (under isoflurane anesthesia) or CO_2_.

### Generation of SBP-1 KO *Pb*NK65 parasites

The *P*. *berghei* NK65 reference line 2168cl2 was used to delete the *sbp1* gene. This reference line expresses GFP-Luciferase under the control of the ama-1 promoter [28]. The *sbp1* gene (PBANKA_1101300) was deleted using the construct as described in De Niz *et al*. (pL2109; pOBconGFP-PbΔSBP1) and using standard methods of transfection, selection and cloning of mutant parasites [52], resulting in the generation of cloned lines of Δ*sbp1* parasites. Cloned line 2559cl2 was used for further studies. Correct integration of the construct into the *sbp1* gene locus on chromosome 11 of parasites of line 2168cl2 was analyzed by Southern analysis of PFG-separated chromosomes as described previously (see S1 Fig) [52]. The presence and absence of the SBP-1 ORF was confirmed with PCR in WT and SBP-1 KO parasites respectively.

### Scoring of disease progression

The parasitemia, body weight and clinical score were evaluated daily starting from day 4 post infection (p.i.). Parasitemia was calculated from blood smears of tail blood stained with 10% Giemsa (VWR, Heverlee, Belgium). Where indicated, infected reticulocytes were differentiated from infected normocytes by their larger and darker appearance, as detailed previously [27]. The clinical score was calculated by evaluating different clinical parameters including social activity (SA), limb grasping (LG), body tone (BT), trunk curl (TC), pilo-erection (PE), shivering (Sh), abnormal breathing (AB), dehydration (D), incontinence (I) and paralysis (P). A score of 0 (absent) or 1 (present) was given for TC, PE, Sh and AB and 0 (normal), 1 (intermediate) or 2 (most serious) for the other parameters. The total clinical score was calculated using the following formula: SA + LG + BT + TC + PE + 3 * (Sh + AB + D + I + P). Mice were euthanized when the clinical score reached 10 or more.

### Dissection and analysis of lung pathology

Mice were euthanized with Dolethal and blood was collected by heart puncture. Bronchoalveolar lavage fluid (BALF) was obtained by pinching off the left lungs, after which PBS (500 μl) was instilled in the large lungs through the trachea with a catheter and withdrawn after 30 s. For flow cytometry experiments the left lungs were not pinched off and 750 μl PBS was instilled in the whole lungs to obtain BALF. This was repeated and both lavages were pooled. Next, a transcardial perfusion with 20 ml PBS was performed. RBCs in BALF were counted to determine lung hemorrhages. The BALF was centrifuged (10 min, 335 g, 4°C) and the protein concentration of the supernatant was determined by Bradford assay (Bio-Rad, Hercules, USA). The weight of the unperfused left lung was determined.

### Retro-orbital puncture and blood biochemistry analyses

In some experiments, mice were anesthetized with 3% isoflurane (Iso-Vet, Dechra, Nortwhich, United Kingdom) before retro-orbital puncture was performed with a heparinized (LEO Pharma, Lier, Belgium) glass capillary tube (Hirschmann-Laborgeräte, Eberstadt, Germany). The collected blood was injected in a cartridge in the Epoc Blood Analysis System (Siemens, Munich, Germany) for biochemical analysis. After the blood collection, mice were euthanized by performing heart puncture under anesthesia with 3% isoflurane.

### Pulmonary leukocyte isolation

Leukocytes were isolated from whole lungs by using the gentle MACS dissociator (Miltenyi Biotec, Leiden, Netherlands) for tissue homogenisation followed by a 30 minutes digestion with collagenase D (2 mg/ml, Roche, Mannheim, Germany) and DNase (40 U/ml, Roche) at 37°C in HEPES buffer (10 mM HEPES-NaOH, 150 mM NaCl, 5 mM KCl, 1 mM MgCl_2_, 1.8 mM CaCl_2_, pH 7.4). Next, another tissue homogenisation cycle was performed with the MACS dissociator and cells were passed through a 70 μm cell strainer (VWR). After centrifugation (7 min, 300 g, RT), a Percoll (GE healthcare, Upsala, Sweden; 100% Percoll buffer: 90% Percoll, 9 mM PBS, 0.01 M HEPES, and 0.005 N HCl) density gradient centrifugation was performed to further purify the leukocytes. Cells were resuspended in 40% Percoll buffer in PBS and added onto a layer of 72% Percoll buffer in PBS. After centrifugation (20 min, 491 g, RT, no break) leukocytes located between the two layers were collected. Cell numbers were counted in a Bürker chamber upon staining with trypan blue (VWR) to exclude dead cells.

### Pulmonary endothelial cell isolation

Dissected lungs were placed in RPMI buffer (RPMI Glutamax [Gibco, Amarillo, TX, USA] + 5% fetal calf serum (FCS) + 1% penicillin/streptomycin + 0.01% β-mercaptoethanol) and were cut to small pieces with scissors. Next, digestion medium (RPMI buffer + 2 mg/ml collagenase D + 0.1 mg/ml DNase I) was added to the lungs prior to an incubation step of 30 min at 37°C. Lung tissue was further homogenized with a syringe and needle and digestion medium was added for another incubation step of 15 min at 37°C. Next, tissue was mechanically homogenized for a second time with syringe and needle. After a centrifugation step (5 min, 708 g, RT), cells were resuspended in 10 mM EDTA and PBS + 2% FCS. Cells were centrifuged (5 min, 708 g, RT) and treated with NH_4_Cl buffer for 3 min at 37°C to lyse RBCs. After passage through a 70 μm cell strainer (VWR) cells were resuspended in PBS + 2% FCS and live cells were counted upon staining with trypan blue.

### Cell staining and flow cytometry

Before surface staining, isolated leukocytes and ECs were transferred to 96 well plates and washed with PBS. Cells were incubated 15 min in the dark at room temperature (RT) with mouse FcR-block (Miltenyi Biotec) together with Zombie Aqua (1/1000, BioLegend, San Diego, CA, USA) for lymphoid immune cells and ECs or Zombie UV (1/1000, BioLegend) for myeloid immune cells to discriminate live from dead cells. Next, cells were washed with PBS + 2% FCS + 2 mM EDTA and stained with monoclonal antibodies diluted in Brilliant stain buffer (BD, Franklin Lanes, NJ, USA) for 20 min in the dark at 4°C. The following monoclonal mouse antibodies were used for leukocyte analysis: anti-CD3 (clone 145-2C11, eBioscience, San Diego, CA, USA), anti-CD8 (clone 53–6.7, eBioscience), anti-CD44 (clone IM7, Biolegend), anti-NK1.1 (clone PK136, eBioscience), anti-CD4 (clone RM4-5, eBioscience), anti-CD62L (clone MEL-14, eBioscience), anti-CD45 (clone 30-F11, Biolegend), anti-CD103 (clone 2E7, eBioscience), anti-CD11c (clone N418, Biolegend), anti-CD64 (clone X54-5/7.1, Biolegend), anti-CD24 (clone M1/69, Biolegend), anti-F4/80 (clone BM8, eBioscience), anti-Siglec F (clone 1RNM44N, eBioscience), anti-Ly6G (clone 1A8, BD), anti-Ly6C (clone AL-21, BD), anti-CD11b (clone M1/70, eBioscience), anti-MHC-II (clone M5/114.15.2, BD), anti-KLRG1 (clone 2F1, BD) and anti-CD127 (clone A7R34, eBioscience). For the analysis of ECs following mouse antibodies were used: anti-CD45 (clone 30-F11, Biolegend), anti-CD40 (clone 1C10, eBioscience), anti-ICAM-1 (clone eBioKAT-1, eBioscience), anti-CD36 (clone HM36, Biolegend), anti-MHC-II (clone M5/114.15.2, Biolegend), anti-CD31 (clone MEC 13.3, BD), anti-MHC-I (clone M1/42, BD), anti-VCAM-1 (clone 429, BD). In the panel to characterize CD8^+^ T cells, cells were intracellularly stained after resuspension in Cytofix/Cytoperm solution (BD Biosciences) with anti-perforin (clone S16009A, Biolegend), anti-granzyme B (clone NGZB, eBioscience) and anti-IFN-γ (clone XMG1.2, Biolegend) mouse antibodies in Perm/Wash buffer (BD Biosciences). Cells were then transferred to FACS tubes, washed with PBS and fixated in 0.4% formaldehyde in PBS.

Fixed cells were analysed on the LSRFortessa X20 (BD Biosciences) equipped with the DIVA software and further analysed with FlowJo software (LLC, V10, Ashland, USA). The gating strategies can be found in the supplementary material (S1 Appendix).

### Determination of parasite accumulation in mice by *in vivo* bioluminescence imaging

Mice were injected i.p. with *Pb*NK65 2168cl2 (WT) and SBP-1 KO *Pb*NK65 2559cl2 (SBP-1 KO) parasites. Both parasites express the fusion protein GFP-luciferase under control of the *ama-1* promotor. *In vivo* imaging of luminescence produced by the luciferase expressing parasites was used to determine the parasite accumulation in whole body and lung region [27]. Mice were anesthetized with 2% isoflurane (Iso-Vet) and received D-Luciferin (150 mg/kg, PerkinElmer, Waltham, USA) subcutaneously in the scruff before full body imaging. Luciferase activity was determined using the IVIS Lumina S5 imaging system (PerkinElmer). Quantitative analysis of bioluminescence of whole body and lung region was performed with Living Image 4.5.5 software (PerkinElmer).

### Production and intravenous injection of schizonts

BALB/c mice were infected with WT or SBP-1 KO *Pb*NK65 parasites. Following heart puncture, the collected blood was purified through a Plasmodipur filter (EuroProxima, Arnhem, The Netherlands) to remove leukocytes. After centrifugation of the filtrate, supernatant was discarded and pellet resuspended in parasite medium (25mM HEPES, 2mM L-glutamine, 5mM glucose, 0.08% sodium bicarbonate, 20% FCS in RPMI [Gibco]). The suspension was transferred to a T150 culture flask (Sigma-Aldrich) and gassed for 1.5 min with a gas mixture (5% CO_2_, 5% O_2_ and 90% N_2_) for overnight incubation at 37°C. The next day, schizonts were isolated by a magnetic-activated cell sorting column (MACS, Miltenyi Biotec, Leiden, The Netherlands). Next, C57BL/6 mice were i.v. injected with 10^7^ or 10^8^ schizonts.

### Quantitative reverse transcription-polymerase chain reaction (qRT-PCR)

RNeasy Mini Kit (Qiagen, Hilden, Germany) was used to extract RNA from left lung after mechanical homogenisation in RLT buffer. After extraction, RNA was quantified and cDNA was synthesized using the High Capacity cDNA Reverse Transcription Kit (Applied Biosystems, Waltham, USA). ABI Prism 7500 Sequence Detection System (Applied Biosystems) was used to perform qRT-PCR reaction on cDNA with specific primers (IDT, Leuven, Belgium, S1 Table) in the TaqMan Fast Universal PCR master mix (Applied Biosystems). The relative mRNA expression was determined as 2^-ΔΔCt^, normalized to the mean 2^-CT^ value of the uninfected control mice and to the 2^-CT^ value of the 18S housekeeping gene.

### Statistical analysis

Statistical analysis was done using the GraphPad Prism software (GraphPad software, San Diego, USA, version 8.3.1). The non-parametric Mann–Whitney U test was used to determine the statistical significance between two groups. P-values smaller than 0.05 were considered statistically significant. P-values were defined as follows: *p < 0.05, **p < 0.01, ***p < 0.001, ****p < 0.0001. To correct for multiple testing, the Holm-Bonferroni method was applied when 4 or more comparisons were made. Unless otherwise specified, each dot represents the result from an individual mouse. Horizontal lines represent group medians. Asterisks without horizontal lines represent significant differences compared to the control group. Horizontal lines with asterisk on top indicate significant differences between groups.

The numerical data used in all figures are included in Data S1.

## Supporting information

S1 Fig Generation and genotyping of SBP-1 KO parasites (clones of line 2559). (A) Schematic representation of the *Pb230p* locus of the reference reporter *P*. *berghei* NK65 parasite 2168cl2 which was used to generate the deletion SBP-1 parasite line. (B) Schematic representation of the generation of the *Pbsbp-1* deletion line. The SBP-1 deletion-construct (pL2109) was used to replace the *Pbsbp-1* coding sequence with the positive selectable marker (SM; *tgdhfr/ts*) cassette, resulting in the generation of the *PbΔsbp-1* (line 2559cl2) after positive selection with pyrimethamine. The construct pL2109 targets the *Pbsbp-1* gene by double cross-over homologous recombination. After genotyping and confirmation of correct construct integration, this line was cloned by limiting dilution. (C) Genotype analysis of clones 1,2,3 and 5 of line 2559 parasites by Southern analysis of chromosomes (chr.) separated by pulsed-field gel electrophoresis (PFGE). Hybridisation of PFG-separated chr. with a probe recognizing the 3’-UTR *Pbdhfr/ts* gene. This probe recognizes the sbp-1 deletion construct pL2109 integrated into the sbp-1 locus (PBANKA_1101300) on chr. 11. In addition it recognizes the endogenous *Pbdhfr/ts* gene on chr. 7 and the GFP-luciferase expression cassette integrated into chr. 3. (D) The genotype of the parasites was determined with PCR analysis. The reference *p28* gene (bands at 400 bp) was present in both 2168cl2 (WT) and 2559cl2 (SBP-1 KO) parasites, assuring adequate DNA quality. The *Pbsbp-1* gene from the 2168cl2 clone was replaced by the h*dhfrxyfcu*-construct through double cross-over recombination. This was confirmed by the detection of the h*dhfrxyfcu*-construct (bands at 1000 bp) only in the SBP-1 KO parasites.
(TIF)

S2 Fig Reduced parasite sequestration in SBP-1 KO infected mice compared to WT infected mice. C57BL/6 mice were infected with WT and SBP-1 KO *Pb*NK65 parasites. Bioluminescence data shown were corrected for parasitemia by dividing the radiance with the peripheral parasitemia. This was done for (A) whole body and (B) lung region in WT and SBP-1 KO infected mice. Horizontal lines with asterisks on top indicate significant differences between groups. Data of two experiments, n = 8–12 per group.
(TIF)

S3 Fig Pulmonary pathology is associated with increased lung weight and hemorrhages. C57BL/6 mice were infected with WT and SBP-1 KO *Pb*NK65 parasites. (A) Infected mice were euthanized and dissected at indicated timepoints and lung weight was determined. (B) Hemorrhages in the lungs were assessed by counting RBCs in BALF. Asterisks above data points indicate significant differences compared to control mice. Data of three experiments, n = 4–14 per group. Mann-Whitney U test with Holm-Bonferroni correction for multiple testing (number of tests = 11) was performed.
(TIF)

S4 Fig Increased spleen weight and lymphoid and myeloid cell number in spleens of SBP-1 KO infected mice. (A) Spleen weight of control, WT and SBP-1 KO infected mice at day 8 p.i.. Flow cytometric analysis at day 8 p.i. of splenic lymphoid and myeloid cells. Absolute cell numbers of (B) CD4^+^ T cells (CD3^+^ NK1.1^-^ CD4^+^), (C) CD8^+^ T cells (CD3^+^ NK1.1^-^ CD4^+^), (D) NK cells (CD3^-^ NK1.1^+^), (E) B cells (CD3^-^ NK1.1^-^ B220^+^), (F) neutrophils (Ly6G^+^ CD11b^+^), (G) eosinophils (Siglec F^+^ CD11c^-^), (H) Ly6C^+^ monocytes (CD11b^+^ MHCII^-^ Ly6C^+^) and (I) Ly6C^-^ monocytes (CD11b^+^ MHCII^-^ Ly6C^-^) are shown. Asterisks above data points indicate significant differences compared to control mice, asterisks above a horizontal line show significant differences between infected groups. Panel A: data of three experiments, n = 16 per group. Panel B-I: data of two experiments, n = 6–12 per group.
(TIF)

S5 Fig Differential expression of cytotoxic markers and KLRG1 in splenic CD8+ T cells and CD8+ T effector cells between WT and SBP-1 KO group. Mean fluorescent intensity (MFI) of perforin, granzyme B, IFN-γ and KLRG1 of (A-D) splenic CD8^+^ T cells and (E-H) CD8^+^ T effector cells at day 8 p.i.. Asterisks above data points indicate significant differences compared to control mice, asterisks above a horizontal line show significant differences between infected groups. Data of two experiments, n = 6–12 per group.
(TIF)

S6 Fig KLRG1 expression is negatively correlated with perforin and granzyme B expression in CD8+ T cells in the lungs of WT and SBP-1 KO infected mice at day 8 post infection. Linear correlation analysis of the MFI of (A) perforin, (B) granzyme B and (C) IFN-γ with MFI of KLRG1 in CD8^+^ T cells of WT (blue dots) and SBP1-KO infected mice (green dots) at day 8 p.i.. Spearman correlation test was performed, r (Spearman correlation coefficient) and p-values are shown. Data of two experiments, n = 10–12 per group.
(TIF)

S1 Appendix Gating strategies for flow cytometry analysis. (PDF)

S1 Table List of primers used for RT-qPCR. (TIF)

S1 Data Numerical data used in figures. The underlying numerical data for Fig panels 1A, 1B, 2B, 2C, 2D, 3A, 3B, 3C, 3D, 3E, 3F, 3G, 3H, 4A, 4B, 5A, 5B, 5C, 5D, 6A, 6B, 6C, 6D, 6E, 6F, 6G, 6H, 6I, 7H, 7I, 7J, 7K, 7L, 7M, 8A, 8B, 8C, 8D, 8E, 8F, 8G, 8H, 8I, 9A, 9B, 9C, 9E, 9G, 9I, 9K and numerical data for Supplementary Figure panel S2A, S2B, S3A, S3B, S4A, S4B, S4C, S4D, S4E, S4F, S4G, S4H, S4I, S5A, S5B, S5C, S5D, S5E, S5F, S5G, S5H, S6A, S6B and S6C.
(XLSX)
