# Supplementary figures and images for: Skeleton binding protein-1-mediated parasite sequestration inhibits spontaneous resolution of malaria-associated acute respiratory distress syndrome
Source: PLoS Pathog. 2021 Nov 29;17(11):e1010114. doi: 10.1371/journal.ppat.1010114 (PMC8659713; doi:10.1371/journal.ppat.1010114)

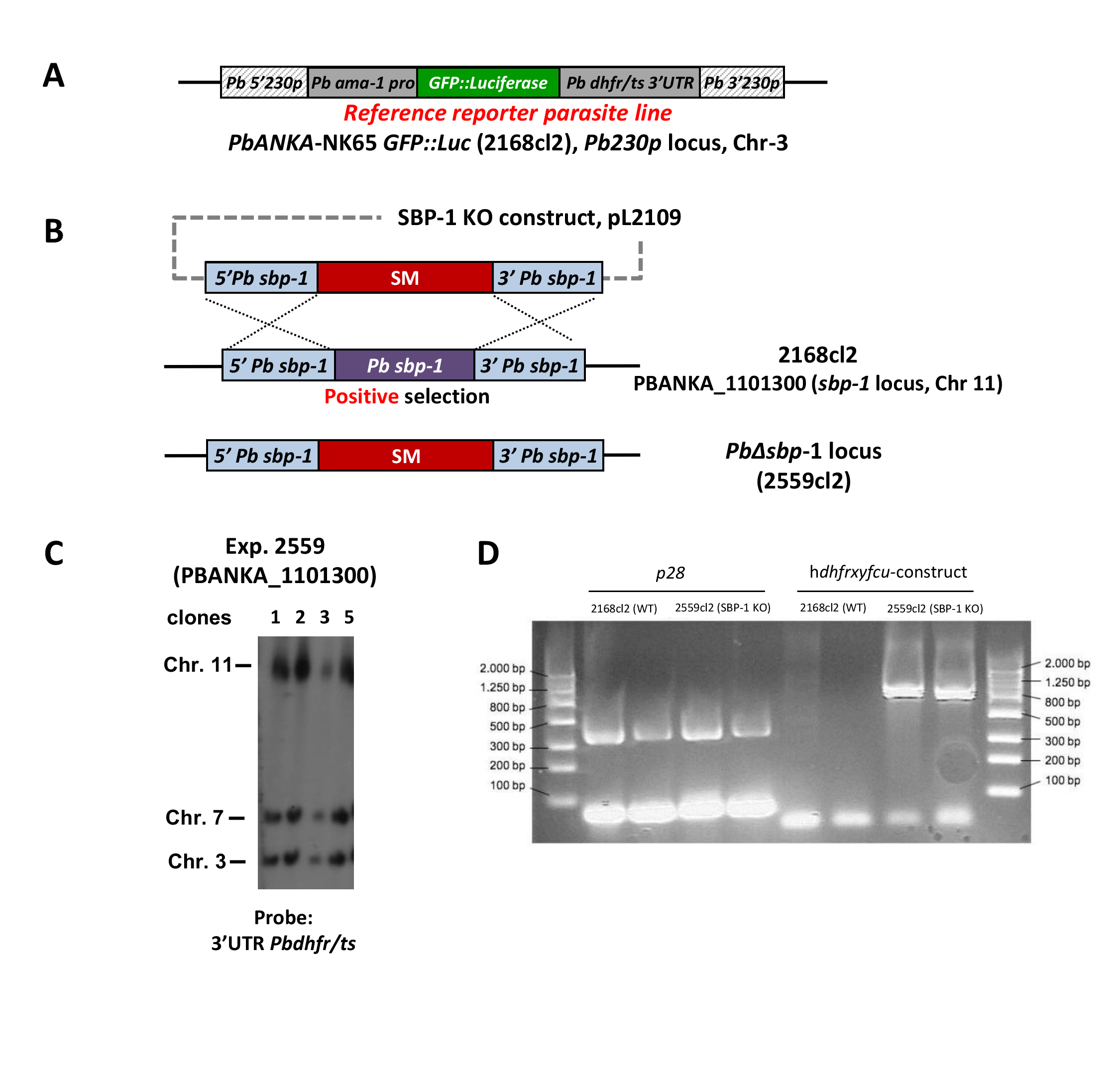

Supplement: S1 Fig — (A) Schematic representation of the Pb230p locus of the reference reporter P. berghei NK65 parasite 2168cl2 which was used to generate the deletion SBP-1 parasite line. (B) Schematic representation of the generation of the Pbsbp-1 deletion line. The SBP-1 deletion-construct (pL2109) was used to replace the Pbsbp-1 coding sequence with the positive selectable marker (SM; tgdhfr/ts) cassette, resulting in the generation of the PbΔsbp-1 (line 2559cl2) after positive selection with pyrimethamine. The construct pL2109 targets the Pbsbp-1 gene by double cross-over homologous recombination. After genotyping and confirmation of correct construct integration, this line was cloned by limiting dilution. (C) Genotype analysis of clones 1,2,3 and 5 of line 2559 parasites by Southern analysis of chromosomes (chr.) separated by pulsed-field gel electrophoresis (PFGE). Hybridisation of PFG-separated chr. with a probe recognizing the 3’-UTR Pbdhfr/ts gene. This probe recognizes the sbp-1 deletion construct pL2109 integrated into the sbp-1 locus (PBANKA_1101300) on chr. 11. In addition it recognizes the endogenous Pbdhfr/ts gene on chr. 7 and the GFP-luciferase expression cassette integrated into chr. 3. (D) The genotype of the parasites was determined with PCR analysis. The reference p28 gene (bands at 400 bp) was present in both 2168cl2 (WT) and 2559cl2 (SBP-1 KO) parasites, assuring adequate DNA quality. The Pbsbp-1 gene from the 2168cl2 clone was replaced by the hdhfrxyfcu-construct through double cross-over recombination. This was confirmed by the detection of the hdhfrxyfcu-construct (bands at 1000 bp) only in the SBP-1 KO parasites. (TIF) [file ppat.1010114.s001.tif]

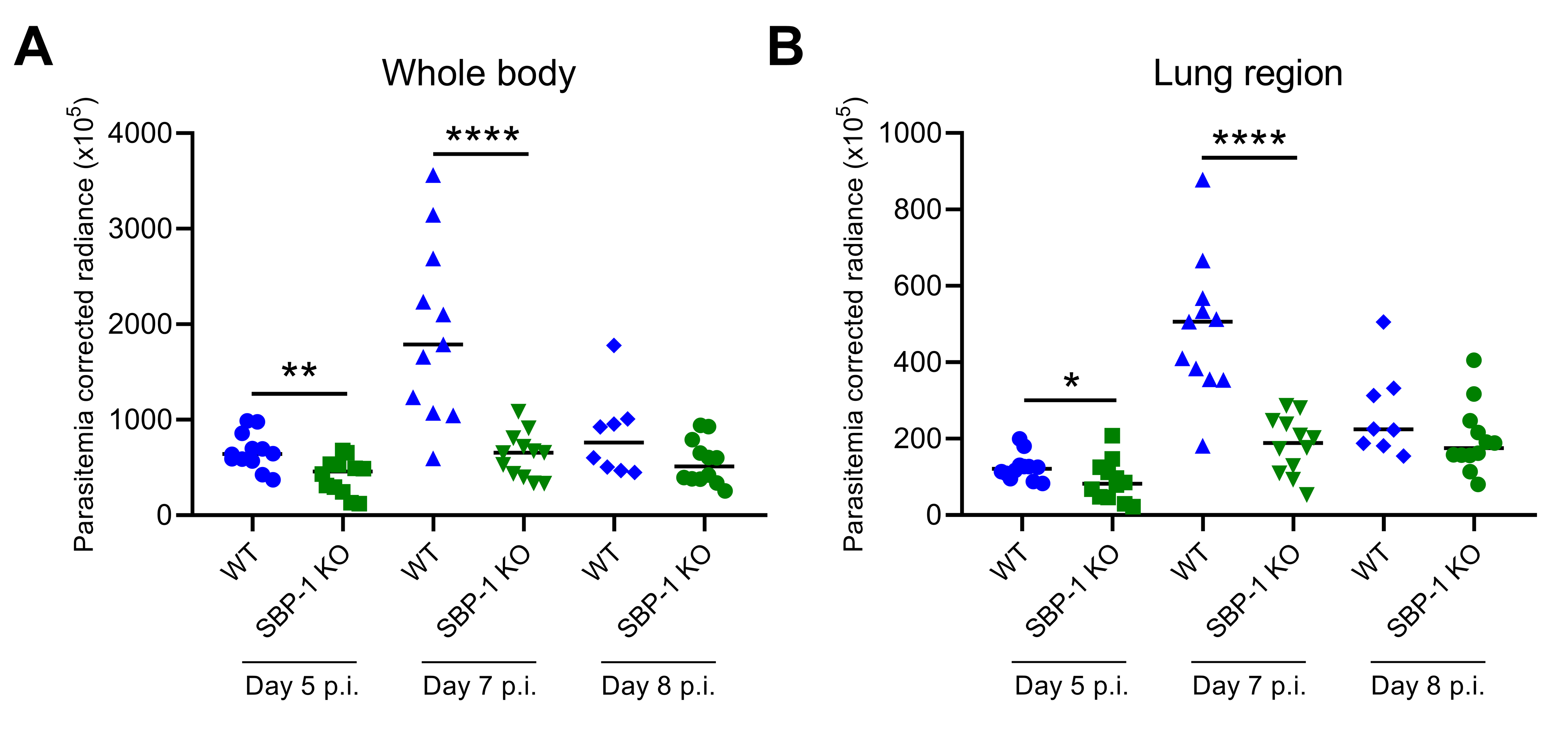

Supplement: S2 Fig — C57BL/6 mice were infected with WT and SBP-1 KO PbNK65 parasites. Bioluminescence data shown were corrected for parasitemia by dividing the radiance with the peripheral parasitemia. This was done for (A) whole body and (B) lung region in WT and SBP-1 KO infected mice. Horizontal lines with asterisks on top indicate significant differences between groups. Data of two experiments, n = 8–12 per group. (TIF) [file ppat.1010114.s002.tif]

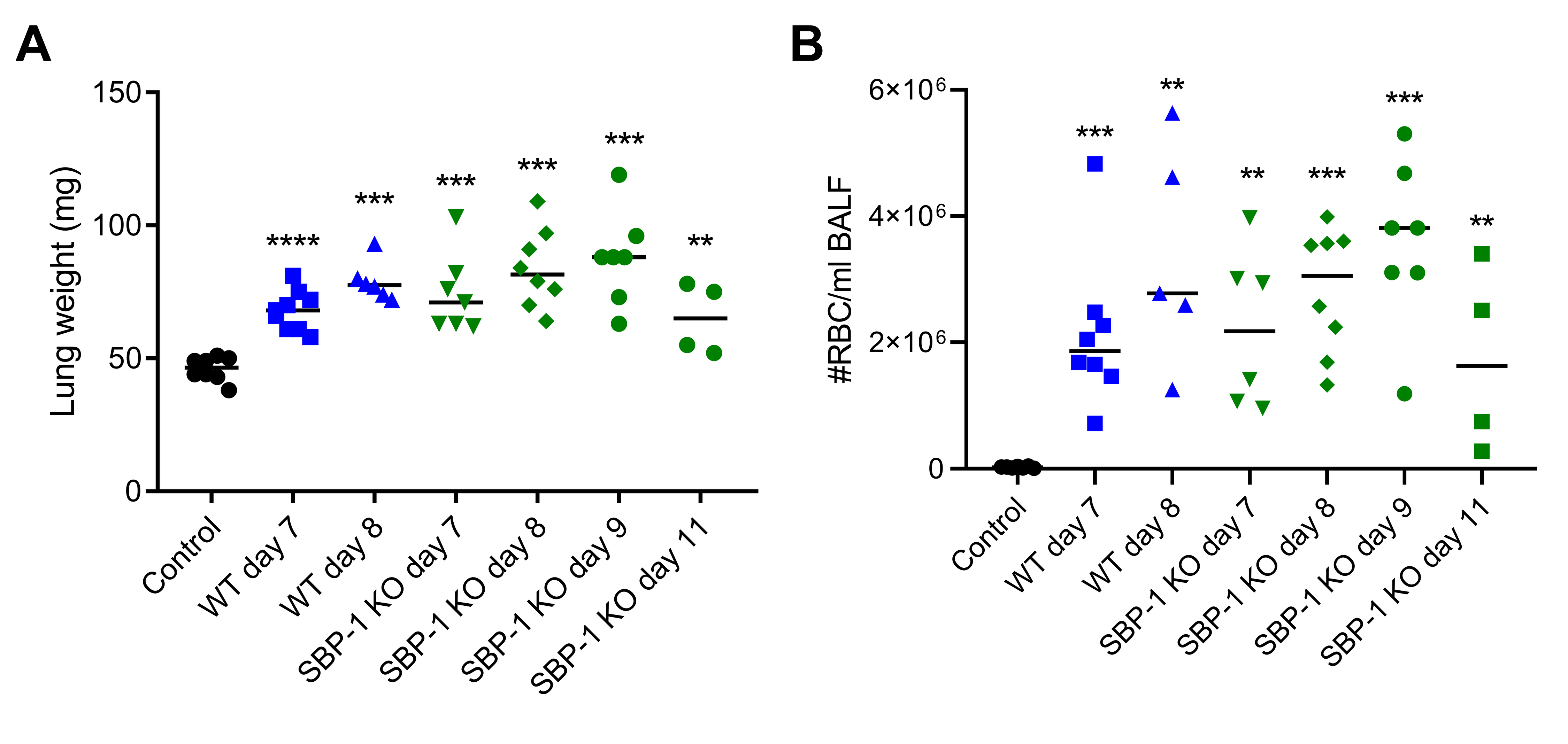

Supplement: S3 Fig — C57BL/6 mice were infected with WT and SBP-1 KO PbNK65 parasites. (A) Infected mice were euthanized and dissected at indicated timepoints and lung weight was determined. (B) Hemorrhages in the lungs were assessed by counting RBCs in BALF. Asterisks above data points indicate significant differences compared to control mice. Data of three experiments, n = 4–14 per group. Mann-Whitney U test with Holm-Bonferroni correction for multiple testing (number of tests = 11) was performed. (TIF) [file ppat.1010114.s003.tif]

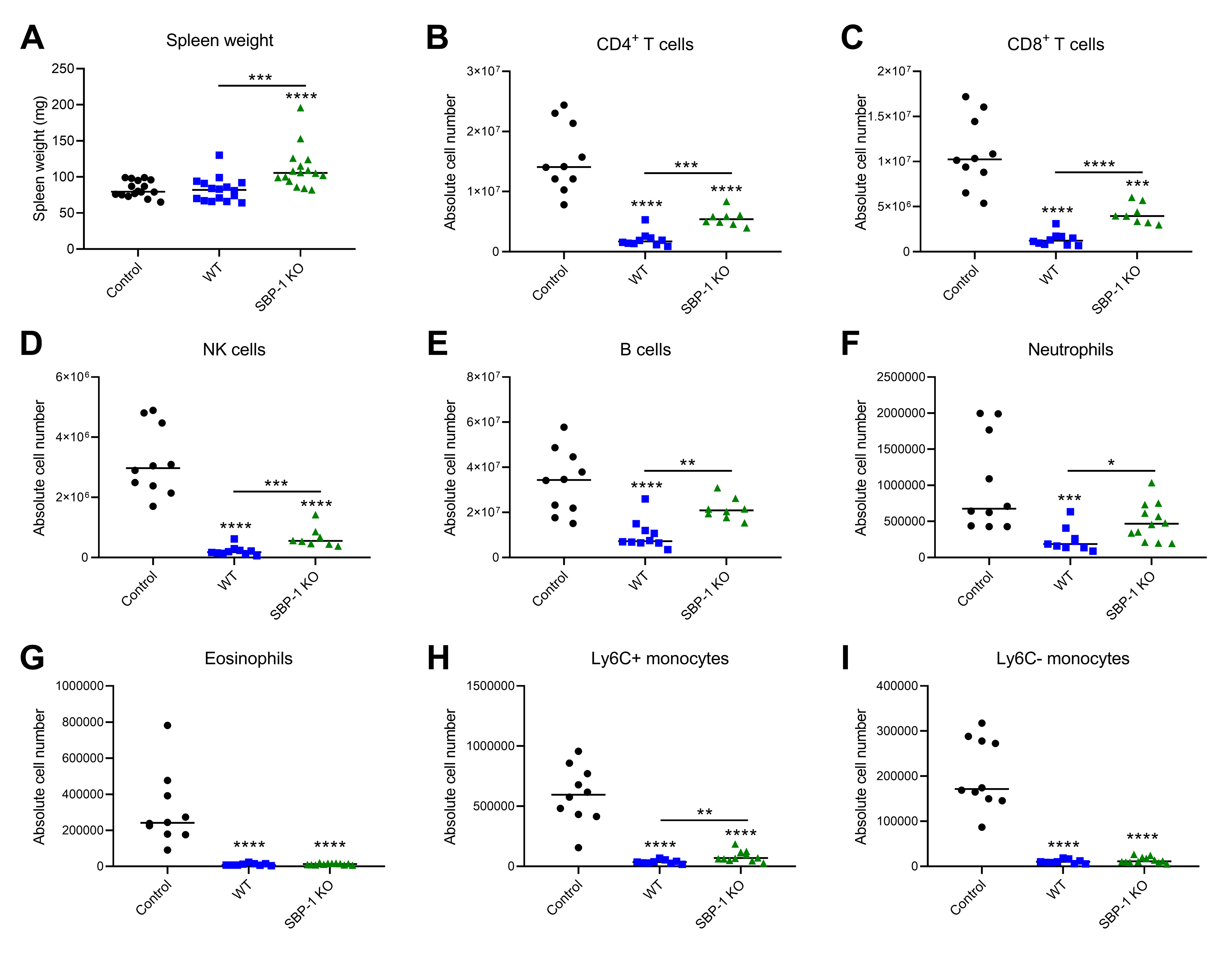

Supplement: S4 Fig — (A) Spleen weight of control, WT and SBP-1 KO infected mice at day 8 p.i.. Flow cytometric analysis at day 8 p.i. of splenic lymphoid and myeloid cells. Absolute cell numbers of (B) CD4+ T cells (CD3+ NK1.1- CD4+), (C) CD8+ T cells (CD3+ NK1.1- CD4+), (D) NK cells (CD3- NK1.1+), (E) B cells (CD3- NK1.1- B220+), (F) neutrophils (Ly6G+ CD11b+), (G) eosinophils (Siglec F+ CD11c-), (H) Ly6C+ monocytes (CD11b+ MHCII- Ly6C+) and (I) Ly6C- monocytes (CD11b+ MHCII- Ly6C-) are shown. Asterisks above data points indicate significant differences compared to control mice, asterisks above a horizontal line show significant differences between infected groups. Panel A: data of three experiments, n = 16 per group. Panel B-I: data of two experiments, n = 6–12 per group. (TIF) [file ppat.1010114.s004.tif]

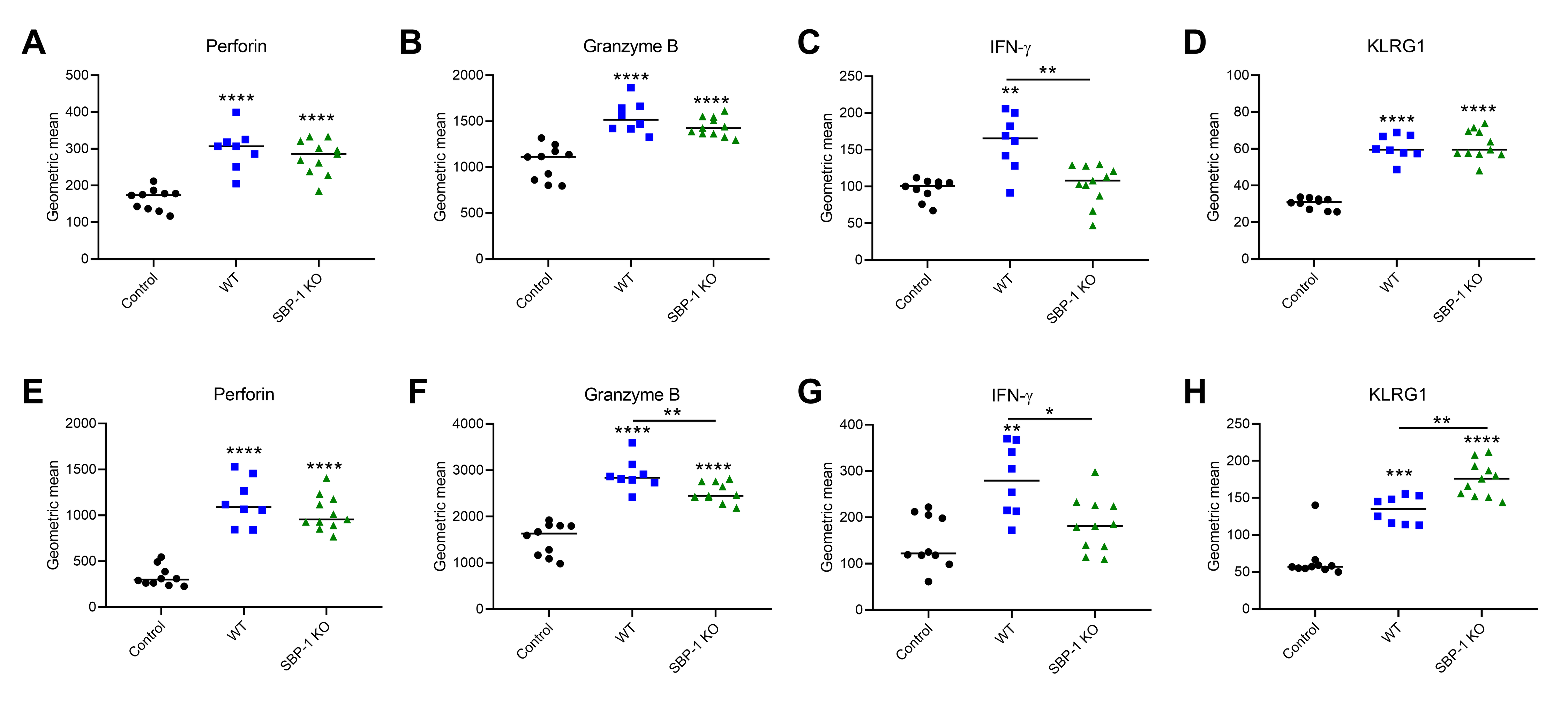

Supplement: S5 Fig — Mean fluorescent intensity (MFI) of perforin, granzyme B, IFN-γ and KLRG1 of (A-D) splenic CD8+ T cells and (E-H) CD8+ T effector cells at day 8 p.i.. Asterisks above data points indicate significant differences compared to control mice, asterisks above a horizontal line show significant differences between infected groups. Data of two experiments, n = 6–12 per group. (TIF) [file ppat.1010114.s005.tif]

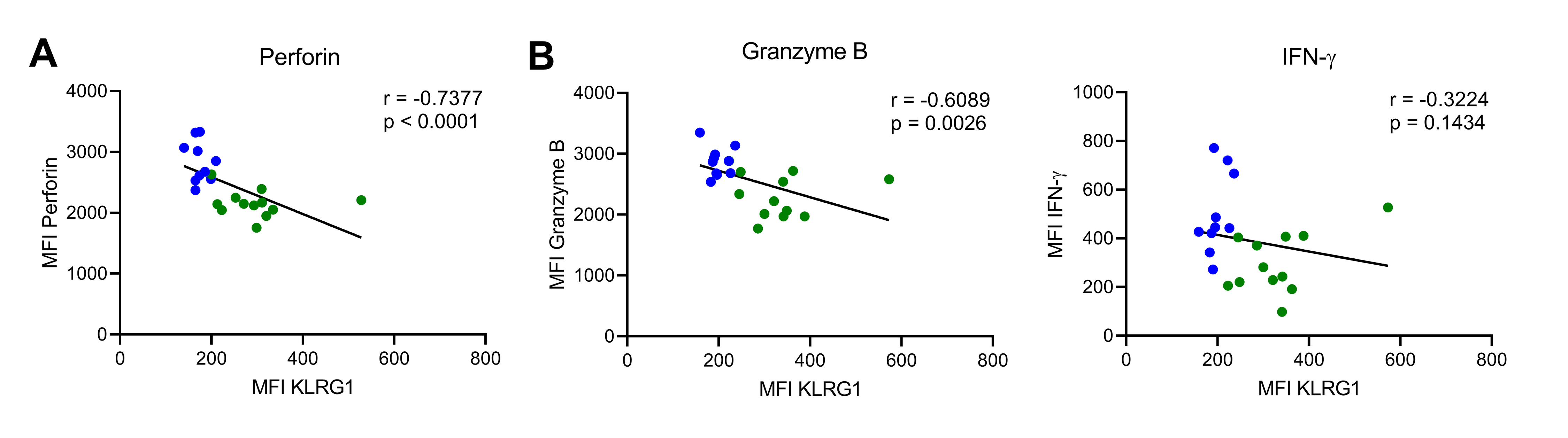

Supplement: S6 Fig — Linear correlation analysis of the MFI of (A) perforin, (B) granzyme B and (C) IFN-γ with MFI of KLRG1 in CD8+ T cells of WT (blue dots) and SBP1-KO infected mice (green dots) at day 8 p.i.. Spearman correlation test was performed, r (Spearman correlation coefficient) and p-values are shown. Data of two experiments, n = 10–12 per group. (TIF) [file ppat.1010114.s006.tif]

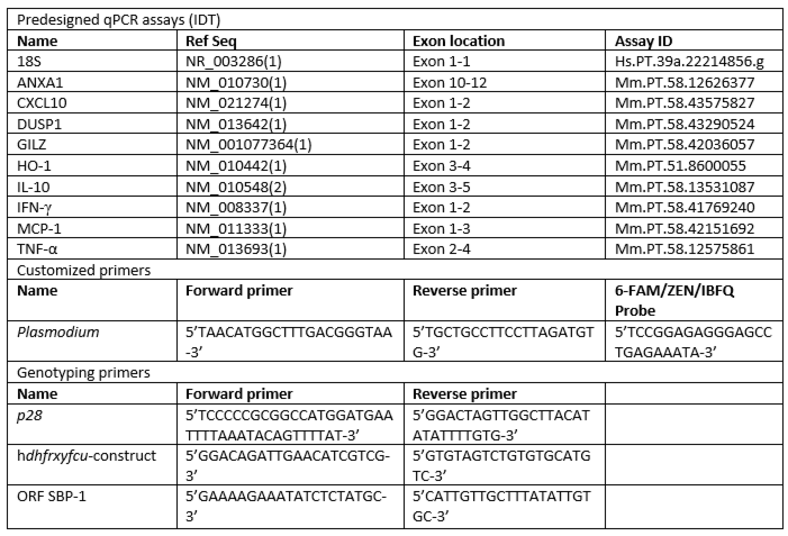

Supplement: S1 Table — (TIF) [file ppat.1010114.s008.tif]
