# Supplementary material for: Skeleton binding protein-1-mediated parasite sequestration inhibits spontaneous resolution of malaria-associated acute respiratory distress syndrome
Source: PLoS Pathog. 2021 Nov 29;17(11):e1010114. doi: 10.1371/journal.ppat.1010114 (PMC8659713; doi:10.1371/journal.ppat.1010114)

S1 Appendix: gating strategies

Lung and spleen cells were isolated and stained for flow cytometry. After exclusion of red blood cells, debris and doublets of cells, all live cells (Live dead negative) were gated.

Lymphoid and myeloid panel

The leukocytes were identified as CD45 positive. For the lymphoid panel in the lungs, the alveolar macrophages, which are autofluorescent, were gated out by plotting Live dead versus FSC-A.

Endothelial cell panel

Endothelial cells in the lungs were identified as CD31 positive and CD45 negative. The mean fluorescent intensity of various markers on the endothelial cells was determined.

$CD8^+$ T cell panel

$CD8^+$ T cells in the lungs were gated as CD8 positive. The mean fluorescent intensity of various markers on the $CD8^+$ T cells was determined.

For each gating strategy, the gating of an uninfected control and an infected mouse at day 8 p.i. is shown below.

Gating strategy of lymphoid panel in lung

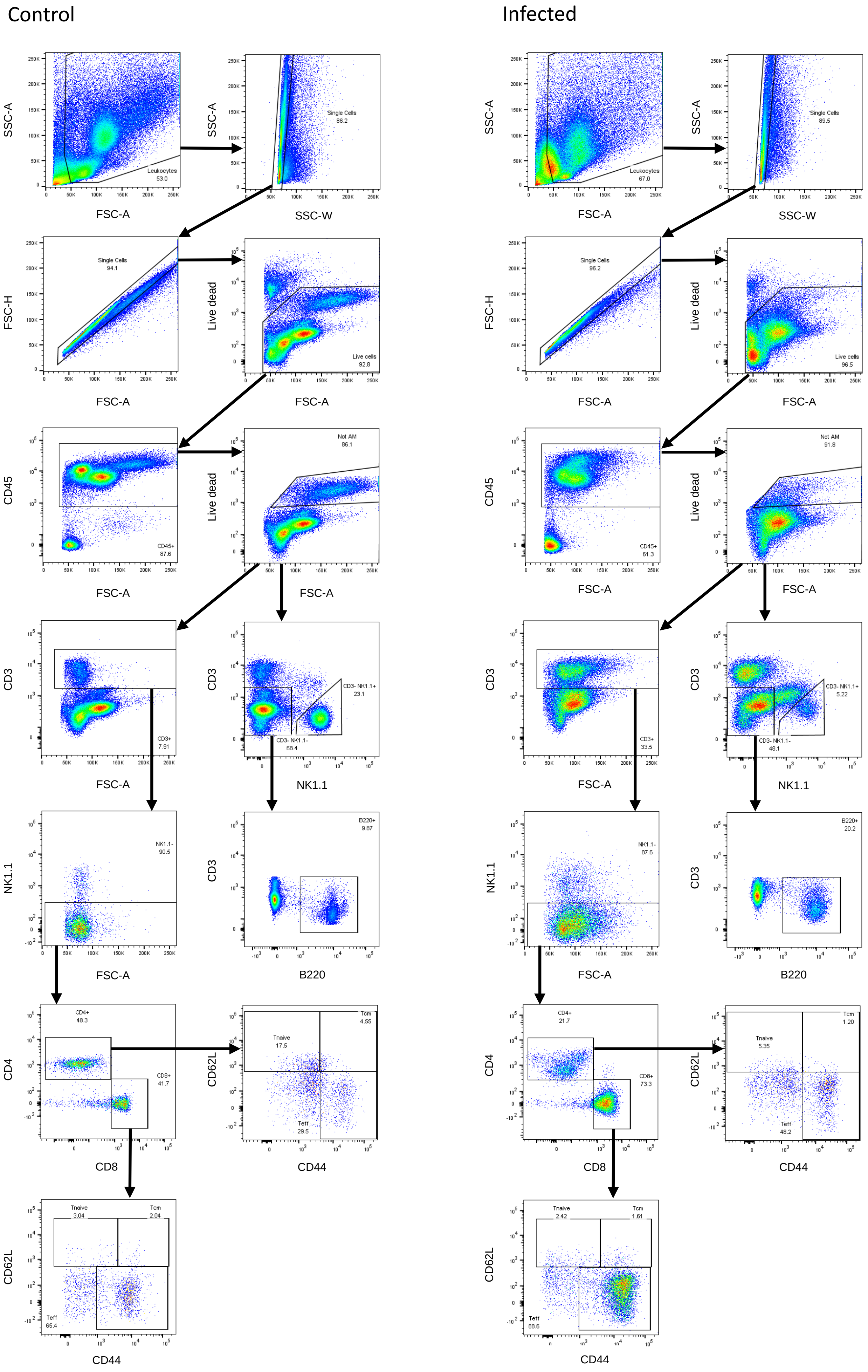

Gating strategy of myeloid panel in lung

Control

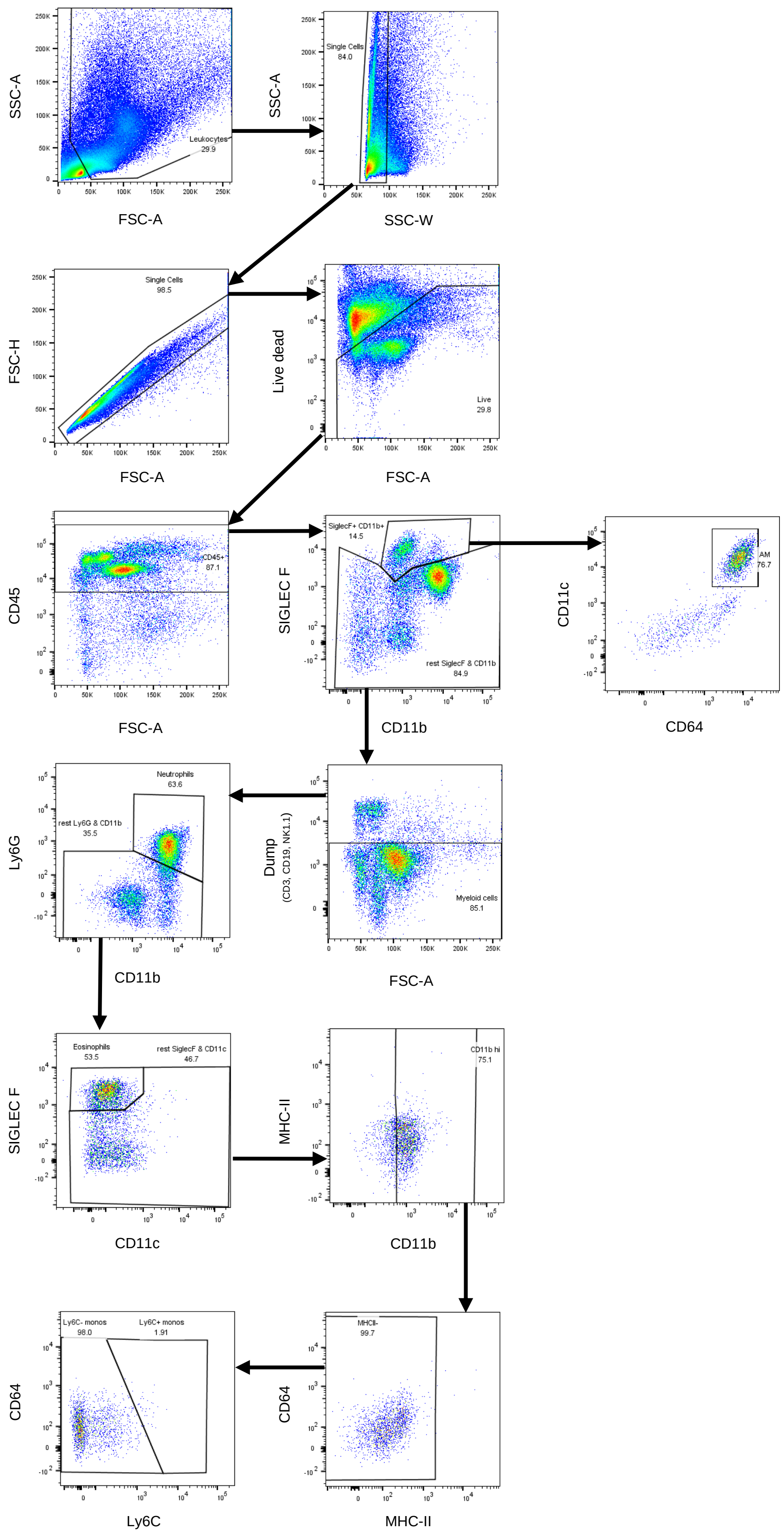

Gating strategy of myeloid panel in lung

Infected

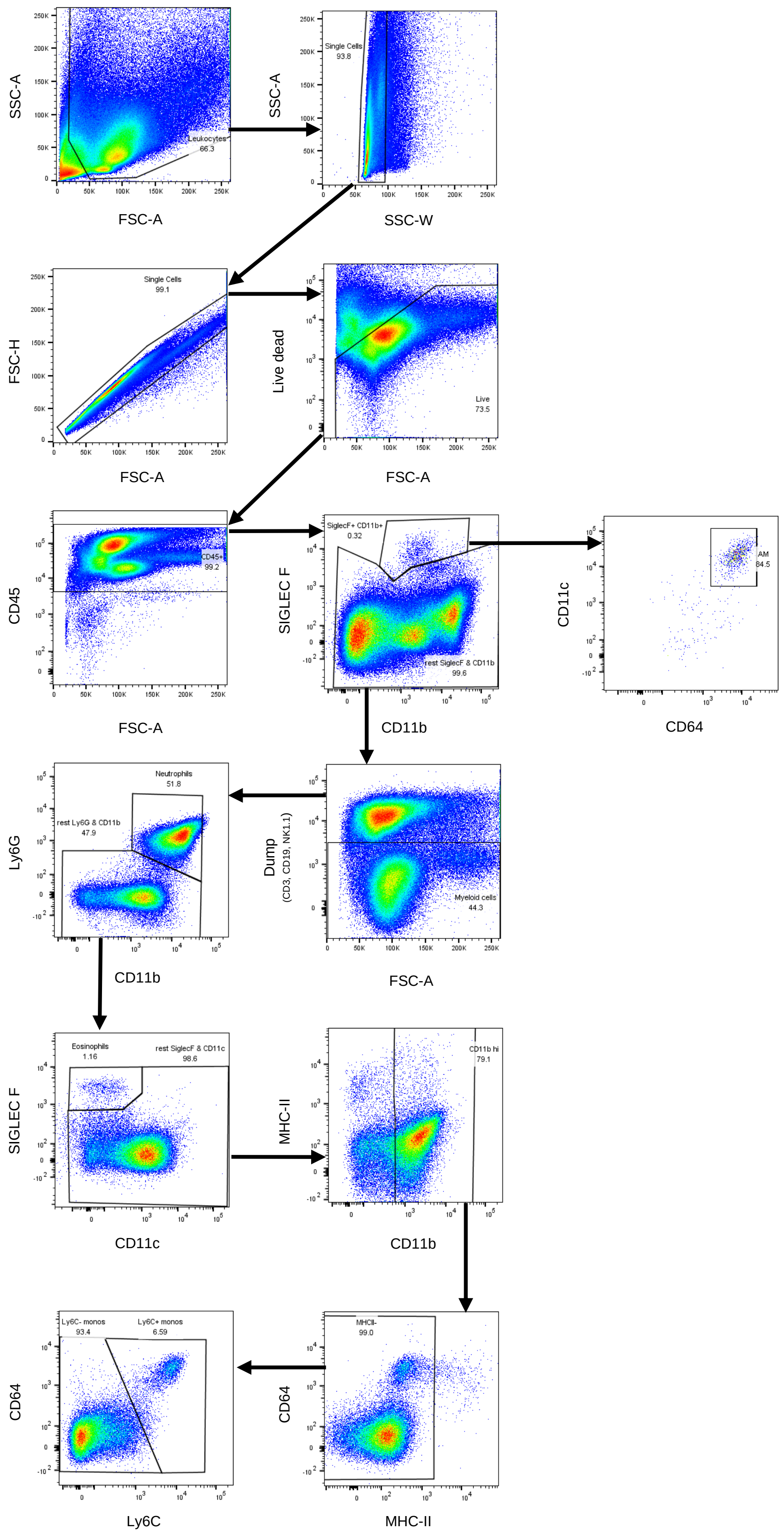

Gating strategy of endothelial cell panel in lung

Control and infected

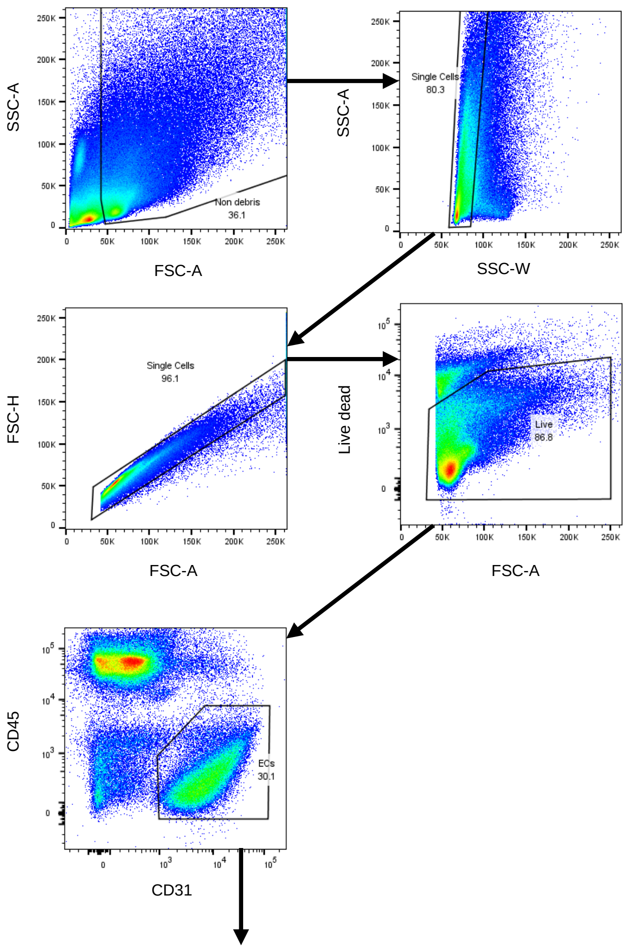

MFI ICAM-1, VCAM-1, MHC-I, MHC-II, CD40, CD36

Gating strategy of CD8$^+$ T cell panel in lung

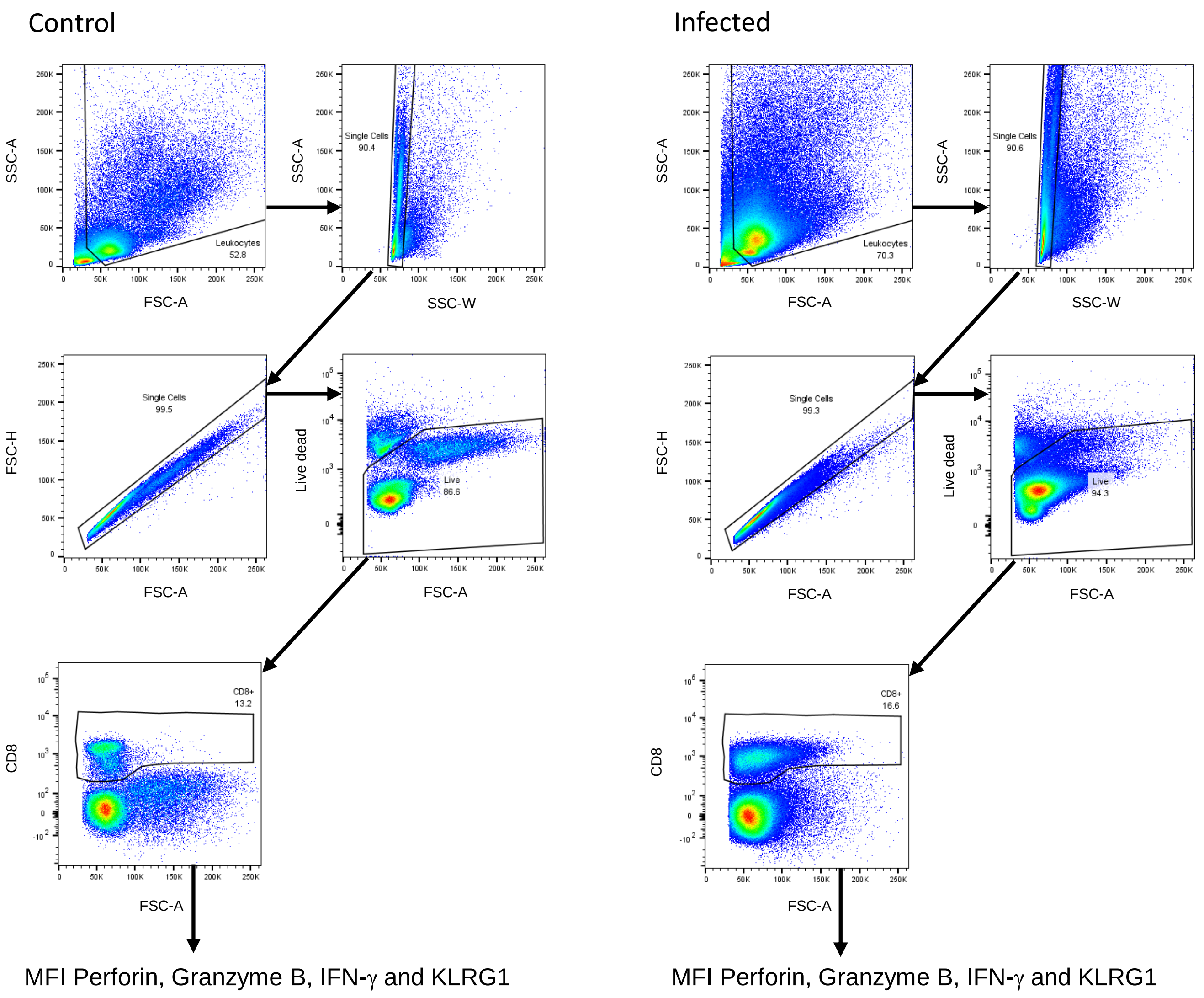

Gating strategy of lymphoid panel in spleen

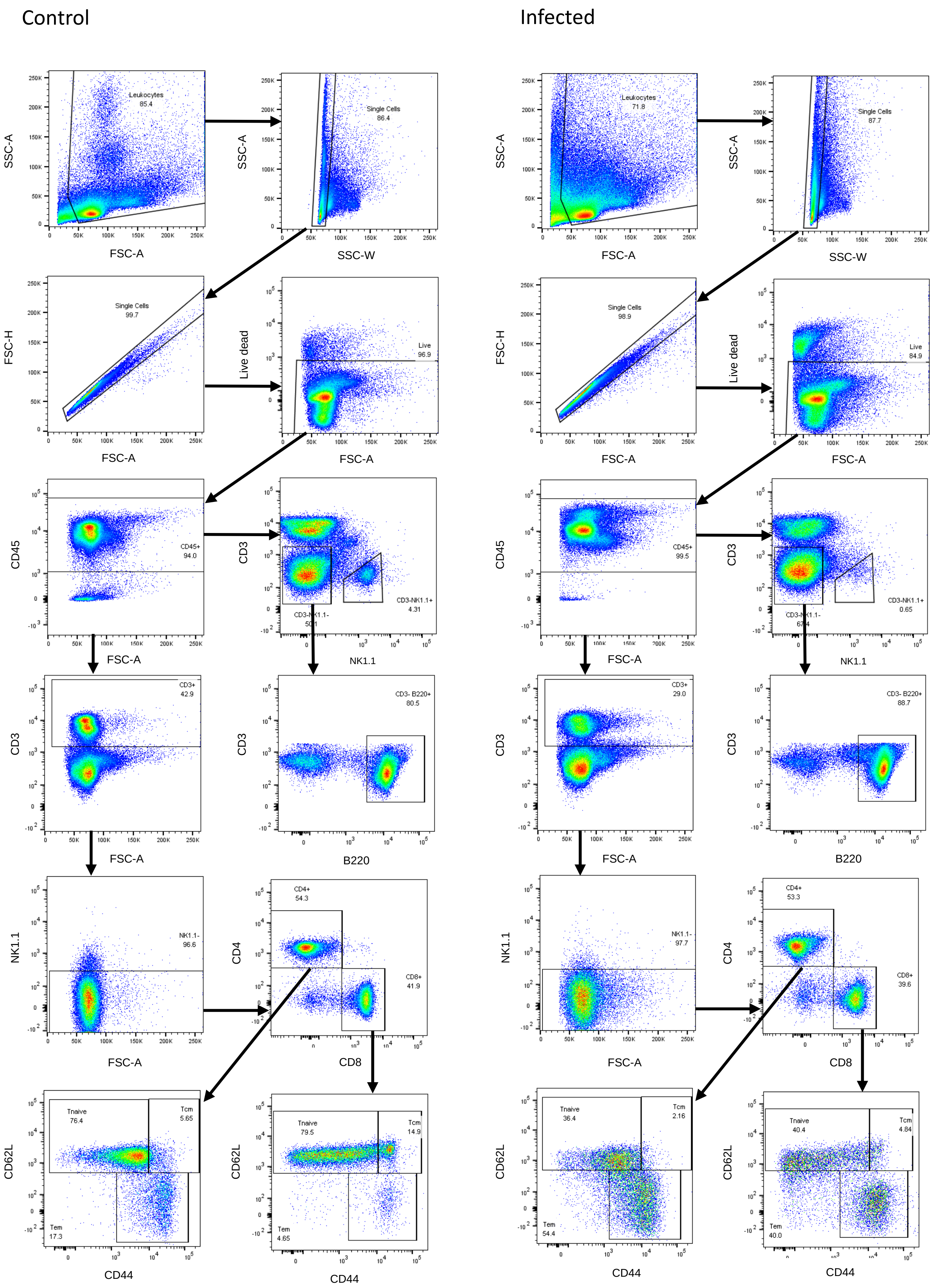

Gating strategy of myeloid panel in spleen

Control

Infected

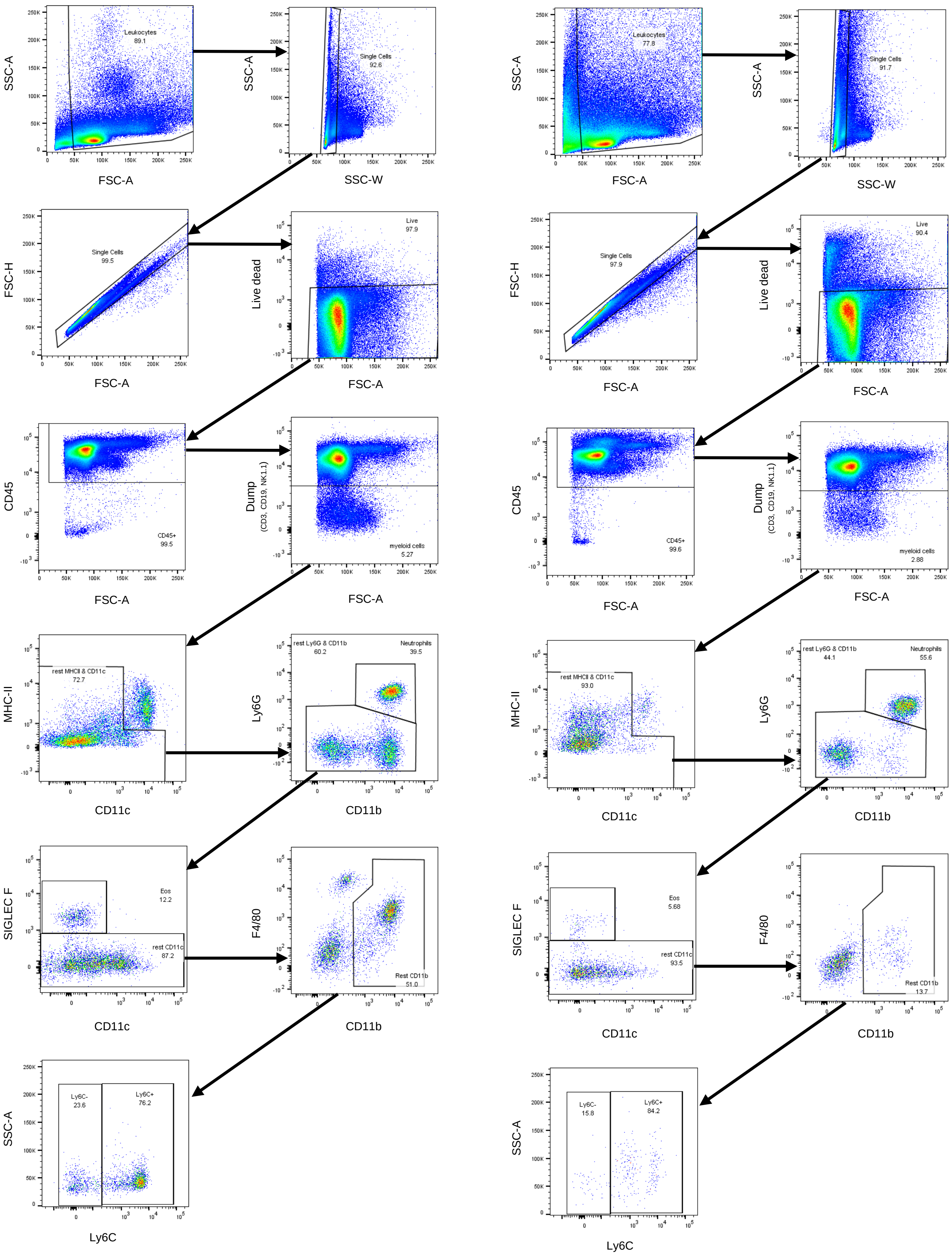

Supplement: S1 Appendix — (PDF) [file ppat.1010114.s007.pdf]
